# Advancing automated identification of airborne fungal spores: guidelines for cultivation and reference dataset creation

**DOI:** 10.1007/s10453-025-09864-y

**Published:** 2025-06-02

**Authors:** Nicolas Bruffaerts, Elias Graf, Predrag Matavulj, Astha Tiwari, Ioanna Pyrri, Yanick Zeder, Sophie Erb, Maria Plaza, Silas Dietler, Tommaso Bendinelli, Elizabet D’hooge, Branko Sikoparija

**Affiliations:** 1 Mycology and Aerobiology, Sciensano, Brussels, Belgium; 2 Swisens AG, Emmen, Switzerland; 3 Institute for Data Science, University of Applied Sciences North Western Switzerland, Windish, Switzerland; 4 Biology Department, National and Kapodistrian University of Athens, Athens, Greece; 5 Federal Office of Meteorology and Climatology MeteoSwiss, Payerne, Switzerland; 6 Environmental Remote Sensing Laboratory, EPFL, Lausanne, Switzerland; 7 Faculty of Medicine, University of Augsburg, Augsburg, Germany; 8 CSEM Alpnach, Centre Suisse d’Electronique et de Microtechnique, Alpnach, Switzerland; 9 BCCM/IHEM, Sciensano, Brussels, Belgium; 10 BioSense Institute - Research Institute for Information Technologies in Biosystems, University of Novi Sad, Novi Sad, Serbia

**Keywords:** Airflow cytometry, Automatic identification, Culture collection, Fungal spores, Machine learning

## Abstract

Airborne bioparticles, including fungal spores, are of major concern for human and plant health, necessitating precise monitoring systems. While a European norm exists for manual volumetric monitoring, there's a growing interest in automated real-time methods. However, these methods rely heavily on machine learning, facing challenges due to diverse particle characteristics and limited training data availability, especially for fungal spores. This study aims to address this gap by outlining best practices for collecting reference material and creating tailored datasets for training algorithms. Using 17 fungal species from the Belgian fungi collection BCCM/IHEM, including five *Alternaria* species, key aspects such as in vitro cultivation, dry spore harvest, and aerosolization were addressed. Simple classification models were developed, achieving varying accuracies on different monitors. The Plair Rapid-E+ demonstrated accuracies ranging from 83.4% to 95.1% (macro average F1-score 0.61), with better recognition for *Cladosporium* spp. and *Curvularia caricae-papayae*. The SwisensPoleno Jupiter, initially achieving a macro average F1-score of 0.77 with holographic images of eight genera, improved to 0.83 when combined with fluorescence data. Accuracies ranged from 55 to 95%, with notable performance for *Alternaria* spp. and *Curvularia caricae-papayae*. Species differentiation was also shown to be possible for *Cladosporium*, but was more difficult for some *Alternaria* species, while the macro average F1-score remained good (0.72). Overall, this protocol paves the way for more efficient, standard, and accurate automatic identification of airborne fungal spores.

**Supplementary Information:**

The online version contains supplementary material available at 10.1007/s10453-025-09864-y.

## Introduction

Bioaerosols impact both human and plant health in various ways, making their monitoring of great interest. Although a norm has been established (EN, 16868, 2019), based on the manual volumetric Hirst method, there is a growing demand from end-users, such as the medical community, allergic individuals, atmospheric modellers, and plant disease managers, for automated monitoring (Tummon et al., 2021, 2024). The diverse characteristics of bioaerosols in terms of size, origin and chemical composition (Després et al., 2012; Fröhlich-Nowoisky et al., 2016) make their monitoring challenging, particularly with respect to automatic real-time identification. Current approaches rely on physical or chemical properties, with some systems even employing a combination of both (Huffman et al., 2020). Despite these advances, challenges remain in differentiating fungal species due to overlapping fluorescence signatures. Studies such as those by O’Connor et al. (2011) and Saari et al. (2015) have highlighted the influence of species type, cultivation conditions, and exposure history on the fluorescence spectra of fungal spores. Moreover, foundational work on the biophysical basis of bioaerosol autofluorescence (Pöhlker et al., 2011; Hill et al., 2013) provides key insight into the molecular contributors to emission signatures detected by these systems.

A recent evaluation comparing several operational bioaerosol monitors against the standard method has shown that airflow cytometry relying on morphological and chemical characterization performs comparably to digital microscopy (Maya-Manzano et al., 2023). A series of instruments relying on laser-induced fluorescence (LIF) and airflow cytometry-based technologies have also been employed extensively to characterize fungal spore composition and dynamics. The UV-APS, as evaluated by Kanaani et al. (2007), has shown sensitivity to the age and air exposure history of fungal spores, influencing fluorescence signal intensity and thus classification accuracy. WIBS systems have undergone substantial refinements for enhanced spectral resolution and particle characterization. For example, Markey et al. (2024) and Crawford et al. (2023) demonstrated the capabilities of WIBS-4 + in distinguishing fungal spores from pollen and other ambient particles under varying meteorological conditions. The BAA500, meanwhile, has recently been used with AI-driven classification approaches to detect Alternaria spores in real-time (González-Alonso et al., 2023).

The quantity and complexity of data gathered automatically underscores the need for advanced data analytic methods to develop effective identification algorithms. Supervised machine learning methods are commonly used in automated systems for bioaerosol classification (Buters et al., 2022; Tummon et al., 2022), mostly relying on reference datasets upon which algorithms can be trained. As with training humans to identify particles by microscopy using reference slides of known material, these algorithms require datasets to be trained with appropriate labels, either generated from reference material or environmental samples. Obtaining such datasets is generally easier for pollen than for fungal spores. For pollen, reference material collection is relatively straightforward because the source plants can often be visually identified in the field with confidence, particularly for well-known anemophilous species. These species tend to produce large amounts of pollen, which facilitates sampling. A common technique involves harvesting mature inflorescences and placing them in paper envelopes or drying chambers, where the pollen is allowed to dehisce naturally from the anthers under ambient or controlled conditions (Hoekstra, 1995). In contrast, reference fungal spore collection requires growing colonies in controlled conditions, with extraction methods often tailored to specific species (Drew Smith et al., 1961).

So far, automatic identification of fungal spores relies on reference data obtained from exceptional events during operational measurements, i.e. when the fungal spore of interest is largely dominant in the air. This approach was applied for *Alternaria* spp. and total airborne spores using different measurement methods such as light microscopy imaging from an impactor (González-Alonso et al., 2023), airflow cytometry with holograms (Erb et al., 2023), and the combination of scattering data and laser-induced fluorescence (Simović et al., 2023). However, applications of this approach are limited by the scarcity of suitable episodes and by the difficulty to efficiently clean operational data. Therefore, the availability of large and clean reference datasets, encompassing the predominant spectrum of airborne fungal spores, is expected to facilitate the automated identification of a broad range of fungal diversity in ambient air, at least as allowed by the standard method (Anees-Hill et al., 2022).

The aim of this study is to outline and describe the best practices for cultivating and collecting reference material, and creating datasets, in order to train algorithms for the classification of airborne fungal spores based on airflow cytometer measurements. Key aspects addressed here include: access to fungal strains whose spores are representative of the outdoor air mycobiome; controlled growing and sporulation of single species cultures; harvesting of clean and individual fungal spores from sporulating colonies; aerosolization of dry spores to challenge automatic monitors; and creation and cleaning of training datasets. In addition, basic classification models have been developed to assess the identification potential.

## Fungal spore production

### Fungal reference material

The standard monitoring method EN 16868, which relies on direct microscopic observation of aerobiological samples, offers limited resolution for assessing the species diversity of airborne fungal spores. This limitation arises in part because aerobiological samples contain a wide range of morphologically similar spores, making fine taxonomic identification difficult without specialized mycological expertise. Moreover, many fungal taxa cannot be reliably distinguished based on spore morphology alone, as accurate identification often requires examination of mycelial structures that are absent in airborne samples. As for indoor mycological monitoring methods, more sensitive culture-based analysis from these aerobiological samples could offer a better representativity of its species diversity and therefore an opportunity to collect reference material. However, such an approach would require substantial effort in cleaning and isolating colonies to ensure only single-species isolates that can be cultivated for spore production. Additionally, given the rapidly changing fungal taxonomy and the consequent necessity to perform DNA identification, it is advisable to use reference strains from certified microorganism culture collections, which ensure reliability and reproducibility.

In this study, 17 certified fungal strains (Table 1), covering 11 different genera, were obtained from BCCM/IHEM (Sciensano, Belgium), a collection of yeasts and moulds of medical and veterinary interest that gathers more than 16,000 strains, representing more than 350 genera and 1,200 species of Ascomycetes, Basidiomycetes and Mucoromycetes. Therefore, the selection of fungal strains was primarily driven by the BCCM/IHEM public catalogue,^1^ which is limited to health-relevant species and their sister taxa and species in the environment. In the future, the evaluation of strains of particular interest to agriculture or forestry (Entomophthoraceae, Pucciniomycetes, etc.) could be explored in collaboration with collections dedicated specifically to these fields.

**Table 1:** List of the 17 fungal strains selected from the BCCM/IHEM fungal culture collection

Label in this study	Current species name	Common synonym name(s)	Strain name
*Alternaria alternata*	*Alternaria alternata* (Fries ex Fries) von Keissler	*Alternaria citri* Ellis & Pierce emend. Bliss & Fawcett *Alternaria destruens* E.G. Simmons *Alternaria soliaegyptiaca* E.G. Simmons *Alternaria tenuis* Nees *Alternaria tenuissima* (Kunzeex Fries) Wiltshire	IHEM 3327
*Alternaria arborescens*	*Alternaria arborescens* E.G. Simmons	–	IHEM 18586
*Alternaria botrytis*	*Alternaria botrytis* (Preuss) Woudenberg & Crous	*Ulocladium botrytis* Preuss *Stemphylium botryosum* var. *ulocladium* Sacc *Stemphylium botryosum* var. *botrytis* (Preuss) Lindau	IHEM 2964
*Alternaria chartarum*	*Alternaria chartarum* Preuss	*Ulocladium chartarum* (Preuss) E.G. Simmons *Alternaria chartarum* f. *stemphylioides* (Bliss) P. Joly *Alternaria stemphylioides* Bliss *Sporidesmium polymorphum* var. *chartarum* (Preuss) Cooke	IHEM 20041
*Alternaria terricola*	*Alternaria terricola* Woudenberg & Crous	*Ulocladium tuberculatum* E. Simmons	IHEM 4136
*Botrytis cinerea*	*Botrytis cinerea* Persoon ex Fries	*Botryotinia fuckeliana* (de Bary) Whetzel *Sclerotinia fuckeliana* (de Bary) Fuckel	IHEM 27814
*Chaetomium globosum*	*Chaetomium globosum* Kunze ex Fries	*Chaetomium rectum* Sergeeva *Chaetomium chartarum* Ehrenb *Chaetomium chlorinum* (Sacc.) Grove *Chaetomium kunzeanum* Zopf *Chaetomium olivaceum* Cooke & Ellis	IHEM 6003
*Cladosporium cladosporioides*	*Cladosporium cladosporioides* (Fresen.) de Vries	*Cladosporium pisicola* W.C. Snyder *Penicillium cladosporioides* Fresen *Hormodendrum cladosporioides* (Fresen.) Sacc	IHEM 26980
*Cladosporium herbarum*	*Cladosporium allicinum* (Fr.) Bensch, U. Braun & Crous	*Cladosporium bruhnei* Linder *Cladosporium herbarum* f. *hordei (*Bruhne) Ferraris *Cladosporium hordei* (Bruhne) Pidopl	IHEM 3260
*Cladosporium sphaerospermum*	*Cladosporium sphaerospermum* Penzig	–	IHEM 25311
*Curvularia caricae-papayae*	*Curvularia caricae-papayae* H.P. Srivast & Bilgrami	–	IHEM 21710
*Epicoccum nigrum*	*Epicoccum nigrum* Link	*Epicoccum purpurascens* Ehrenberg & Schlechtendahl	IHEM 3433
*Exserohilum rostratum*	*Exserohilum rostratum* (Drechsler) Leonard & Suggs emend. Leonard	*Drechslera rostrata* (Drechsler) Richardson & Fraser *Helminthosporium halodes* Drechsler *Helminthosporium leptochloae* Nisikado & Miyake *Helminthosporium rostratum* Drechsler *Bipolaris rostrata* (Drechsler) Shoemaker	IHEM 3524
*Fusarium culmorum*	*Fusarium culmorum* (W.G. Smith) Saccardo	*Fusisporium culmorum* Wm. G. Sm	IHEM 3323
*Fusarium pseudocircinatum*	*Fusarium pseudocircinatum* O'Donnell & Nirenberg	*–*	IHEM 18653
*Pithomyces chartarum*	*Pseudopithomyces chartarum* (Berk. & M.A. Curtis) Jun F. Li, Ariyaw. & K.D. Hyde	*Pithomyces chartarum* (Berk. & M.A. Curtis) M.B. Ellis *Leptosphaerulina chartarum* Cec. Roux, Trans. Br. mycol *Piricauda chartarum* (Berk. & M.A. Curtis) R.T. Moore *Sporidesmium bakeri* Syd. & P. Syd *Sporidesmium chartarum* Berk. & M.A. Curtis	IHEM 15222
*Stemphylium vesicarium*	*Stemphylium vesicarium* (Wallr.) E.G. Simmons	*Stemphylium herbarum* E.G. Simmons *Stemphylium sedicola* E.G. Simmons *Helminthosporium vesicarium* Wallr *Pleospora denotata* (Cooke & Ellis) Sacc *Pleospora pisi* (Sowerby) Fuckel	IHEM 3105

Each strain is listed with its current species name alongside a list of common synonyms, and the designation utilized in this study

The overall abundance of fungal species in the outdoor air was taken into account for the strain selection as fungal spores and their mycelium fragments constitute up to 11% of atmospheric bioaerosols (Tordoni et al., 2021). *Cladosporium* and *Alternaria* are commonly known as the most abundant fungal genera in the ambient air as reported by morphological analyses and eDNA metabarcoding (Banchi et al., 2020; Tordoni et al., 2021), as well as *Epicoccum* and *Stemphylium* are also relatively abundant. It should be noted that routine aerobiological analysis following the standard method EN 16868 relies on spore identification based on their morphology, which usually limits identification up to the level of genus, often denoted as fungal spore type (Galan et al., 2021). The four previously mentioned fungal spore types are routinely measured by many European aerobiological monitoring networks (Anees-Hill et al., 2022), together with *Botrytis*, *Chaetomium*, *Curvularia*, *Drechslera* (*Helminthosporium*), *Pithomyces*, *Torula* and the Aspergillaceae family (*Penicillium*/*Aspergillus* spp.). All these taxa are relevant from the health point of view and were included in this study when possible. A strain of *Exserohilum rostratum* was selected to represent *Drechslera,* a morphologically classified spore type common in aerobiological analysis, as well as *Pseudopithomyces palmicola* for *Pithomyces*. The *Torula* strain tested in this study failed to achieve a satisfactory level of sporulation under the experimental conditions, thereby yielding insufficient material for analysis. In contrast, cultures of *Penicillium chrysogenum* and *Aspergillus fumigatus* strains exhibited prolific growth, facilitating the collection of high spore amounts with minimal hyphal contamination. This was evidenced by the material readily falling onto the glass wall of the slant tube. A gentle inversion onto the bench is enough to dislodge it from the culture surface, making a more complex harvesting system, as described in this study, unnecessary. Furthermore, the majority of spores in this family are smaller than 5 µm and spherical-shaped. Hence, they present less interest when developing algorithms relying on holography data comparatively to other spore taxa. The Aspergillaceae taxon was accordingly excluded from this study despite its availability in the BCCM/IHEM catalogue.

The *Alternaria* taxonomic group is broad and morphologically diverse, and it formally encompasses several taxa that are still commonly identified as separate entities in aerobiological monitoring, such as *Pleospora*, *Ulocladium*, and *Stemphylium*. Morphological species recognition in the *Alternaria* group is based on the branching patterns of conidial chains and on the morphological characteristics of conidia (Simmons et al., 2007). For this reason, in this study, we selected five strains belonging to different species and with contrasted conidia shapes, from spores with a beak (e.g., *A. tenuissima* and *A. arborescens*) to *Ulocladium*-like spores with non-apparent beak (*A. botrytis* and *A. terricola*). Recent molecular systematics have prompted the reassessment of species-group organization within the genus. As a result, certain species names have been subject to change, such as *A. tenuissima* being synonymized with *A. alternata*. Despite differences in conidia colour and beak size, these alterations have been supported (Woudenberg et al., 2015).

Regarding the genus *Cladosporium*, which is one of the largest genera of dematiaceous hyphomycetes, strains representative of three major species complexes were selected, namely *C. cladosporioides* (belonging to the *C. cladosporioides* species complex), *C. sphaerospermum* (*C. sphaerospermum* species complex) and *C. allicinum* (*C. herbarum* species complex). Although these species may be identified thanks to their surface ornamentation (Bensch et al., 2015), holography images of current real-time airflow cytometers are not expected to allow efficient classification within this genus, as for the standard method by light microscopy.

Finally, the genus *Fusarium* comprises a large number of human and plant pathogenic species, for example the *F. fujikuroi* species complex. A strain of *F. pseudocircinatum*, belonging to this complex, was selected for this study. However, the 25–50 µm long, fusiform macroconidia are abundantly mixed with ellipsoidal to cylindrical 5–10 µm microconidia (Ajmal et al., 2022), which may impede the creation of quality training data with uniform spore types. Therefore, a strain of *F. culmorum* was added in the selection. This last species is known to produce macroconidia without microconidia. Comparison of both strains would highlight the potential interference of mixed spore types within a taxon-specific training dataset.

### Culture conditions for sporulating colonies

All freeze-dried stock products were inoculated in slant tubes, following the BCCM/IHEM instructions of use (BCCM/IHEM, 2015), in order to reactivate the growth capacity of the strain. Then, the use of Petri dishes is recommended for subcultures to increase the sporulating surface and ease the access for harvesting. Here, 9 cm diameter Petri dishes (Greiner Bio-One, Belgium) were used. The size of the dish will determine the time required for complete confluence of the culture, which may affect growing dynamics and the sporulating phenotype. If the culture is left under confluence for too long, hyaline mycelium progressively grows on an upper layer (the so-called secondary mycelium).

Although the literature contains optimal culture media for specific fungi groups (Atlas et al., 1993), we limited the set of broad spectrum media to Potato Dextrose Agar (PDA),^2^ diluted Sabouraud (S10)^3^ or V8 agar (V8) (5% V8 juice (Campbell Soup Co.), 1 g MgSO_4_·7H_2_O, 1 g KH_2_PO_4_, 20 g Pastagar, 1 mL oligoelements for 1 L at pH 5.5), for all strains as detailed in Table 2. Sporulation yields were also tested on cultures grown on Malt Extract Agar^4^ and were similar (not shown). In general, the media mentioned here above should be suitable for most species, with the exception of V8 which is more commonly used for *Alternaria* species. V8 is reported to support abundant sporulation and reproducible sporulation patterns that are comparable to those found in nature (Simmons et al., 2007).

**Table 2:** Relative score for ease of harvesting by suction with the cyclone collector (from 0 for the lowest yield to 3 for the highest yield), and microscopically significant presence of hyphae and/or other particles in the harvested sample (Y: presence, N: absence). PDA: Potato Dextrose Agar; S10: Diluted Sabouraud

Species	Culture medium used	Ease of harvesting score	Presence of hyphae/other particles
*Alternaria alternata*	PDA	0	Y
*Alternaria arborescens*	PDA, S10, V8	2	N
*Alternaria botrytis*	PDA	2	N
*Alternaria chartarum*	PDA	1	N
*Alternaria terricola*	PDA	1	N
*Botrytis cinerea*	S10	0	N
*Chaetomium globosum*	S10	1	Y
*Cladosporium cladosporioides*	S10	2	N
*Cladosporium herbarum*	S10	2	Y
*Cladosporium sphaerospermum*	S10	2	N
*Curvularia caricae-papayae*	S10	1	N
*Epicoccum nigrum*	S10	1	N
*Exserohilum rostratum*	S10	1	N
*Fusarium culmorum*	S10	3	N
*Fusarium pseudocircinatum*	S10	1	N
*Pithomyces chartarum*	S10	3	N
*Stemphylium vesicarium*	S10	1	N

Inoculation was performed either by cutting the initial culture into 3–5 agar pieces of 2 × 2 mm, loaded with fungal material, and aseptically placing it on the surface of the Petri dish, or by rubbing one of these pieces by striation over the entire surface of the Petri dish in order to ensure an even growth and sporulation over the whole surface (Li et al., 2020). All dishes were incubated without humidity control at 25 °C in the dark for about two weeks. It is recommended to avoid sealing Petri dishes, as the accumulation of water condensate may distort colonies and sporulation patterns. An incubation period of 5–10 days typically suffices to achieve a satisfactory sporulation yield, although sporulation may occur a few days earlier if inoculation is conducted via striation. The yield of sporulation can be assessed by examining the surface coverage of colourless mycelium. Subsequent to the incubation period, cultures of the 17 selected strains underwent both macroscopic (Supplementary Fig. 1) and stereoscopic inspections (Supplementary Fig. 2).

Notably, all strains exhibited satisfactory levels of sporulation (Supplementary Fig. 1), obviating the need for further optimization of culture conditions like exposure to near-UV light or the use of more specific media (Su et al., 2012). Specifically, *Alternaria* spp. grew rapidly on artificial nutrient media covering the Petri dish surface with densely felted, dark olive brown to black colonies. Mycelium was partly submerged within the agar layer and partly aerial, in particular for the tested *Alternaria alternata* strain. Mature conidia germinated producing secondary mycelium in *A. alternata*, *A. botrytis* and *A. chartarum*. The three *Cladosporium* species produced slow growing, velvety, dark olive colonies with immersed stromatal hyphae. *Botrytis cinerea* covered the entire Petri dish surface with loosely floccose, mouse grey colonies. *Curvularia caricae-papayae*, *Epiccocum nigrum*, *Pithomyces chartarum* exhibited medium growth rate with colonies thinly floccose, black coloured. *Chaetomium globosum* spread rapidly, with sparse white to buff aerial hyphae when young, then without aerial hyphae, becoming greenish olivaceous owing to the aggregation of ascomata. *Exserohilum rostratum* was also relatively fast growing, covering the plate with radial fibery mycelium growth and dark brown colonies. *Stemphylium vesicarium* colonies developed relatively more slowly, having mostly white mycelium and sporulating sparsely. Finally, *Fusarium* spp. covered the entire Petri dish surface rapidly with colonies velvety, radiate, with relatively sparse aerial mycelium, white or reddish.

In general, all tested species, except *Chaetomium globosum*, produced conidia on conidiophores. *Alternaria alternata*, *A. arborescens*, *A. botrytis*, *A. chartarum*, *A. terricola* and *Cladosporium cladosporioides*, *C. herbarum*, *C. sphaerospermum* produced conidia in branched chains while *Curvularia caricae-papayae*, *Stemphylium vesicarium*, *Epicoccum nigrum*, *Pithomyces chartarum* and *Exserohilum rostratum* produced spores singly at the top of simple, short conidiophores. *Botrytis cinerea* and *Fusarium culmorum* and *F. pseudocircinatum* had more complex, branched conidiophores whereas *Chaetomium globosum* produced ascospores inside perithecia that have hairy hyphae around the ostiole. The diverse modes of sporulation and mycelium quantity can be observed in Supplementary Fig. 2. Although sporulation yields may vary across different Petri dishes, culturing a larger number of replicates can mitigate this effect. Phenotypic variations can arise due to differences in micro-environmental conditions during culture and the inherent stability of the strain.

## Fungal spore harvesting and aerosolization

### Isolation from sporulating colonies

In fungal taxonomy studies, a spectrum of methodologies are employed to either collect material for fungal inoculation or describe the morphology of spores (Senanayake, 2020). To enhance yield, spores are typically dislodged from colonies either through washing or by brushing and vacuuming onto a filter or into a cyclone particle collector (Drew Smith et al., 1961; Pogner et al., 2024). For airflow-based extraction, the location of fungal colonies relative to the airstream significantly impacts the number and trajectory of dispersed spores, emphasizing the importance of spatial configuration during lab experiments (Li et al., 2020). Methods that rely on washing spores from sporulating colonies with liquid (such as flooding and subsequent scraping or vortexing to detach spores from conidiophores) are not ideal when analysing spores for specific molecules, as these could be released and dissolved in water. Indeed, biogenic secondary organic aerosols exhibiting their own autofluorescence properties may be subject to aqueous extraction and hence interfere with the fluorescence-based measurements of primary biological aerosol particles like fungal spores (Zhang et al., 2021). Furthermore, this method often leads to significant hyphal contamination (Jacques et al., 2021). Therefore, for creating reference material for airflow cytometry, dry extraction appears most suitable, as it is anticipated to maintain spores unaltered, akin to their natural emission and atmospheric dispersion processes, while also facilitating easier aerosolization.

For certain fungi, such as those belonging to the Aspergillaceae family, spores detach easily and can be dislodged from the colony in substantial quantities. However, for many others, particularly when cultured in vitro, considerable force is necessary to detach and extract spores from the intricate network of hyphae. Brushing is a commonly employed method for extracting dry spores (Jacques et al., 2021), but it often inadvertently collects hyphae along with the spores. Hence, suctioning spores directly from the colony appears to be the optimal approach.

**Fig. 1:**
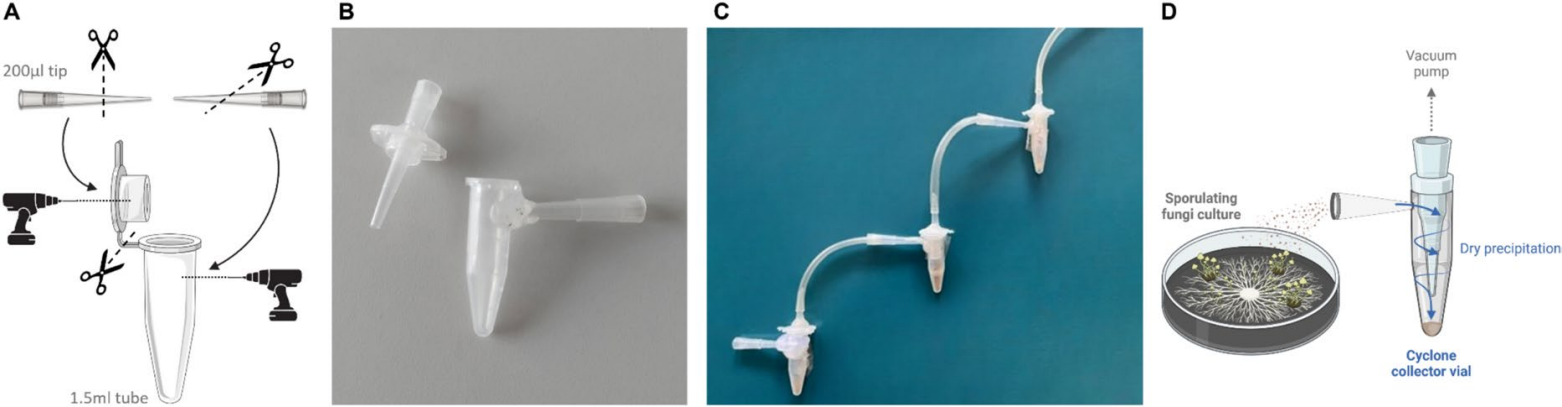
**A** Instructions for constructing a plastic cyclone collector vial using a micro-tube and two micropipette tips, **B** Picture of an assembled cyclone collector vial, **C** Picture of serially connected three cyclone collector vials for increasing the harvesting efficiency of large particles, **D** Scheme of the harvesting system to aspirate dry spores from fungi culture on Petri dish

We opted to vacuum-extract spores from the colony using a cyclone collector (Fig. 1A) rather than a filter to prevent them from embedding in the filter matrix. To simplify fungal spore collection and keep it cost-effective, we aimed to use disposable cyclone collector vials. With this approach, separate cyclone collectors could be used for each sample. We constructed a collector using a single 1.5 mL Eppendorf vial and two 2–200 µL pipette tips (Fig. 1B). One tip is positioned tangentially through the wall of the vial, while the other is inserted through the lid of the vial; both securely affixed using hot plastic glue. Alternatively, this type of collector vial could be fabricated through 3D printing techniques.

The experiments confirmed that cyclone sampling is suitable for efficient extraction of fungal spores from sporulating colonies. Spore extraction varied in ease across different samples (Table 2; Supplementary Video 1), sometimes requiring surface scratching with the cyclone collector tip. It is noteworthy to emphasize that extracting spores from moist colonies (due to condensation on the Petri dish lid or exudate on hyphae) was not possible. Therefore, we ensured that excess moisture was evaporated by leaving the Petri dish lid opened inside the biosafety cabinet at room temperature until it was sufficiently dry, as confirmed under stereomicroscope.

We empirically determined that a flow rate into the cyclone collector exceeding 5 L/min, corresponding to a pump inlet pressure of approximately 100 mmHg, provided sufficient force to detach spores from colonies while generally minimizing the co-harvesting of hyphae. To establish this, we began at the lowest flow rate permitted by our laboratory pump and gradually increased the pressure. This stepwise approach allowed us to identify the minimum critical pressure at which visible and consistent spore collection occurred across all selected taxa. Only once this threshold was reached did we proceed to score both spore yield and hyphal contamination. While this approach was not designed to formally quantify harvesting efficiency across the entire pressure gradient, it enabled us to identify a robust operating point suitable for most species tested. In practice, it was difficult to avoid contact between the cyclone collector tip and aerial hyphae for most species. In some instances, however, such contact facilitated spore release; nevertheless, it occasionally led to an increased collection of hyphae. This phenomenon was particularly notable for *Cladosporium herbarum* and *Chaetomium globosum*, where the delicate structures producing spores tended to fragment, resulting in the harvest of small pieces along with some hyphae. Additionally, increasing the airflow speed by narrowing the inlet could enhance spore detachment. Nevertheless, it is important that the inner diameter of the tube leading from the inlet to the body of the collector does not increase since it would result in deposition of particles on the inside walls, especially concerning small spores. It should be noted that the used cyclone collector vials have limited efficiency for capturing small particles, leading to a notable number of spores escaping and being drawn in by the pump. Efficiency could be substantially increased by connecting multiple cyclones in series (Fig. 1C). Despite this enhancement, the escape of some particles remains inevitable. Thus, it is essential to cover the pump outlet with a filter or place it within a biosafety cabinet to prevent environmental contamination with spores. It is also worth noting that sampling hyaline spores poses a challenge due to their transparent nature, making it difficult to visually confirm their accumulation at the bottom of the collector vial.

To verify the quality of the collected sample (i.e., the quantity of spores and the presence of mycelium), a portion of the material was transferred to a microscopic slide mounted with lactophenol cotton blue and examined via light microscopy (Supplementary Fig. 3). The presence of hyphal fragments was also verified, as expected in particular for species like *Chaetomium globosum* or *Cladosporium herbarum*.

In summary, a relative score (0 to 3) was defined for each tested strain to evaluate the ease of harvesting using the cyclone collector (Table 2). *Botrytis cinerea* and *Alternaria alternata*, cultured respectively on S10 and PDA, received a harvesting score of 0, indicating the lowest yield, and the *A. alternata* sample was also found to contain hyphae and/or other particles. Harvested samples of *Cladosporium herbarum* and *Chaetomium globosum* also contained hyphae fragments, as expected by the unavoidable contact of the cyclone collector tip with aerial mycelium. Conversely, *Fusarium culmorum* and *Pithomyces chartarum* cultured on S10 received the highest harvesting score of 3, signifying optimal harvest efficiency, and with no observable presence of hyphae or other particles in the harvested sample.

### Aerosolization

So far, no standardized method exists for aerosolizing fungal spores due to their size, which often exceeds the capacity of both commercial and custom-made devices utilizing wet or dry dispersion techniques (Lieberherr et al., 2021). Several spore nebulization systems rely on a chamber-aerosol generator optimized for fungal materials (Healy et al., 2012; Scheermeyer & Agranovski, 2009). Achieving a homogeneous suspension of spores in the air, emitted at a steady rate, is crucial when exposing samples to airflow cytometers to prevent clogging and saturation of the detector (Tummon et al., 2022). Conventional aerosolization techniques, such as spraying into a chamber to protect against contamination (Šaulienė et al., 2019), fail to meet the requirement of generating a minimum of 5000 representative particles per taxon, essential for the development of robust identification algorithms (Tummon et al., 2022). To address this challenge, we tested two methods: the SwisensAtomizer (Swisens AG) and a custom-made alternative. Both methods ensured the creation of appropriate training datasets for our study and the same cyclone collector used during the spore harvesting phase was repurposed for the aerosolization phase. This choice aimed to avoid additional container transfer, minimize costs of consumables, and mitigate potential risk of sample contamination and loss of biological material.

#### Exposure to the SwisensPoleno Jupiter

**Fig. 2:**
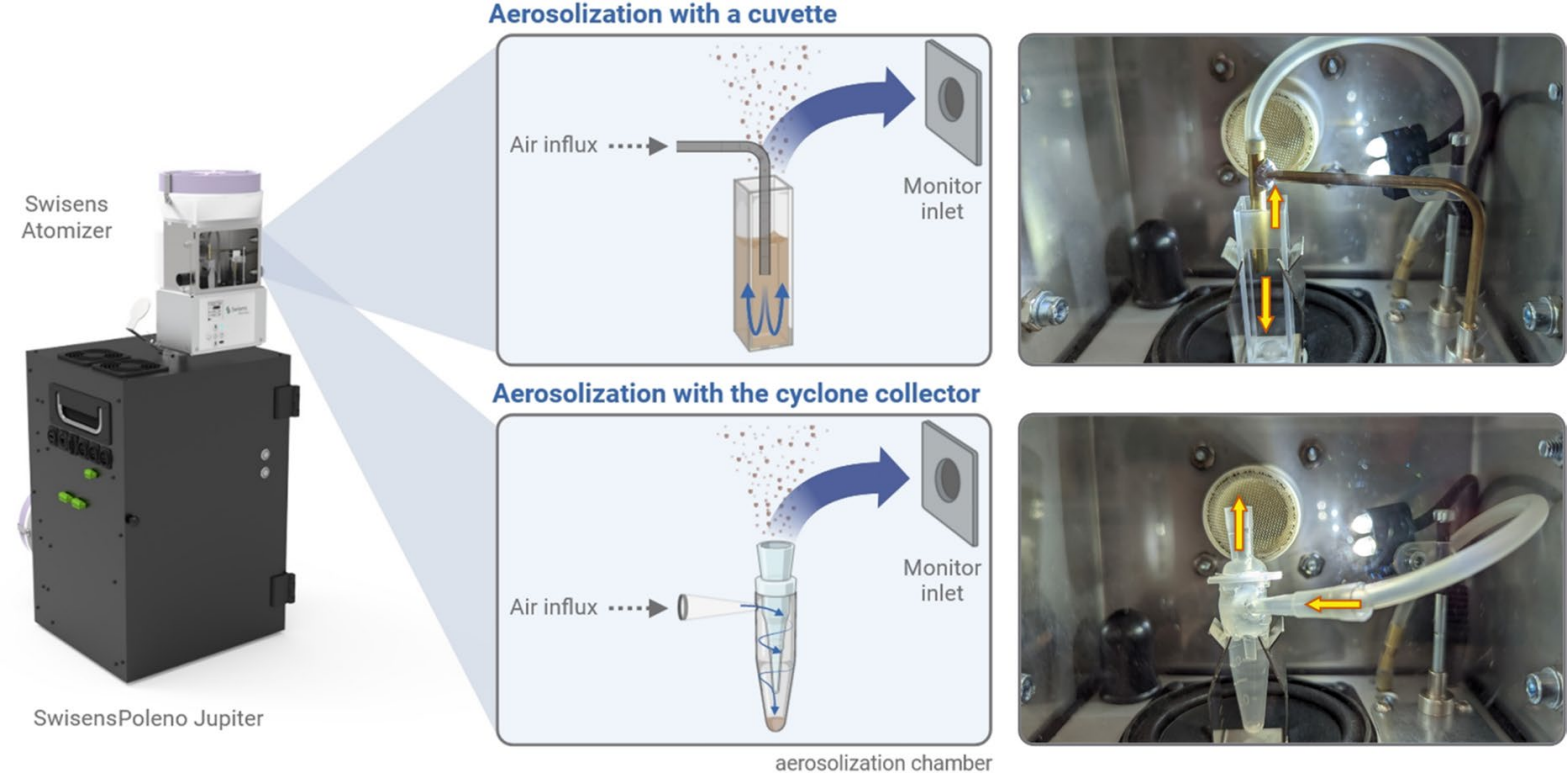
Experimental laboratory setup for the aerosolization of fungal spores and their detection by the SwisensPoleno Jupiter. Homogenous aerosolization within the chamber of the SwisensAtomizer can be performed either by using an open square cuvette or the cyclone collector vial, filled with dry spore material. Arrows indicate the direction of air flows to and out of the container

The airflow cytometer SwisensPoleno Jupiter (Swisens AG) was installed within a biosafety cabinet, where it was exposed to the fungal spores sampled by vacuuming into cyclone collectors. The spores were aerosolized using the SwisensAtomizer. Thus, the sample container was placed in the device on a membrane vibrating at adjustable frequencies and amplitudes, and air was simultaneously blown in the container at chosen intensity. Aerosolization of spores was evaluated with two different containers; an open square cuvette or the cyclone collector previously used for spore harvesting (Fig. 2).

In terms of aerosolization dynamics, the cyclone collector outperformed both the open square cuvette and an open Eppendorf vial, due to its superior airflow stability. The cyclone design likely allows for more consistent airflow patterns, creating a spiral motion that effectively"wipes"the vial wall, aiding in the detachment of particles that may be electrostatically adhered, while the same time due to created centrifugal force keeps majority of particles at the bottom of the container allowing only for limited controlled release of the most loose ones. For some sticky spores, additional aid was required to overcome the effect of centrifugal force. Therefore, an elongated metal bead was inserted into the cyclone collector to further facilitate in detaching spores from the vial wall and fragmenting spore’s aggregates via the vibrations of the system. This consistent airflow resulted in more stable release and detection by the instrument, ensuring reliable and continuous operation. As a result, the instrument operated at a stable detection rate of approximately 300 particles per minute, yielding a sufficient number of particles within a relatively short time frame (Table 3).

**Table 3:** Yield of aerosolization and exposure to the SwisensPoleno Jupiter for each fungal spore species, as evaluated by a relative score (from 0 for the lowest yield to 3 for the highest yield) and by the percentage of exploitable holography imaging data

Species	Ease of aerosolization score	Duration of aerosolization [min]	Total number of particles	Fully recorded particles (two holographic images) [%]
*Alternaria alternata*	1	61	8827	62
*Alternaria arborescens*	1	91	29,785	78
*Alternaria botrytis*	3	39	9447	75
*Alternaria chartarum*	0	35	10,737	88
*Alternaria terricola*	2	60	17,331	85
*Botrytis cinerea*	1	38	12,579	65
*Chaetomium globosum*	3	18	4345	71
*Cladosporium cladosporioides*	1	108	9115	50
*Cladosporium herbarum*	0	15	3867	62
*Cladosporium sphaerospermum*	1	24	4050	60
*Curvularia caricae-papayae*	3	73	12,317	56
*Epicoccum nigrum*	3	35	5748	64
*Exserohilum rostratum*	2	41	9604	69
*Fusarium culmorum*	1	35	9334	61
*Fusarium pseudocircinatum*	1	22	2113	60
*Pithomyces chartarum*	2	29	5405	83
*Stemphylium vesicarium*	1	58	7463	71

The SwisensPoleno Jupiter employs an aerosol concentrator consisting of a three-stage virtual impactor. It directs particles to the measurement volume centre and reduces the airflow through the measurement channel by a factor of approx. 1000, while applying a size dependent separation rate below 10 μm, enabling the high continuous input airflow of 40 L/min. Particle detection is facilitated through the measurement of scattered light by particles crossing two line lasers (450 nm, 685 nm) with a photo-diode. The analysis of measured intensity peaks is used to determine the particle velocity for accurately timing subsequent measurements, while their height is compared to a predefined threshold to set the minimal particle size measured. Two particle images are captured by means of digital inline holography with two orthogonally placed cameras. Following image capture, geometrical particle properties are computed. Thereafter, fluorescence characteristics are quantitatively assessed across five wavebands—ranging from 333 to 694 nm—using LEDs at 280 nm and 365 nm, and a 405 nm laser diode for excitation. The use of holographic images and fluorescence spectrum have previously proven successful for pollen identification (Sauvageat et al., 2020 and Erb et al., 2023) and will therefore be used again. To mitigate variances such as particle position within the measurement channel and slight intensity differences in the excitation sources, fluorescence spectra are normalized relative to the sum of measured intensities per source (Erb et al., 2023).

**Fig. 3:**
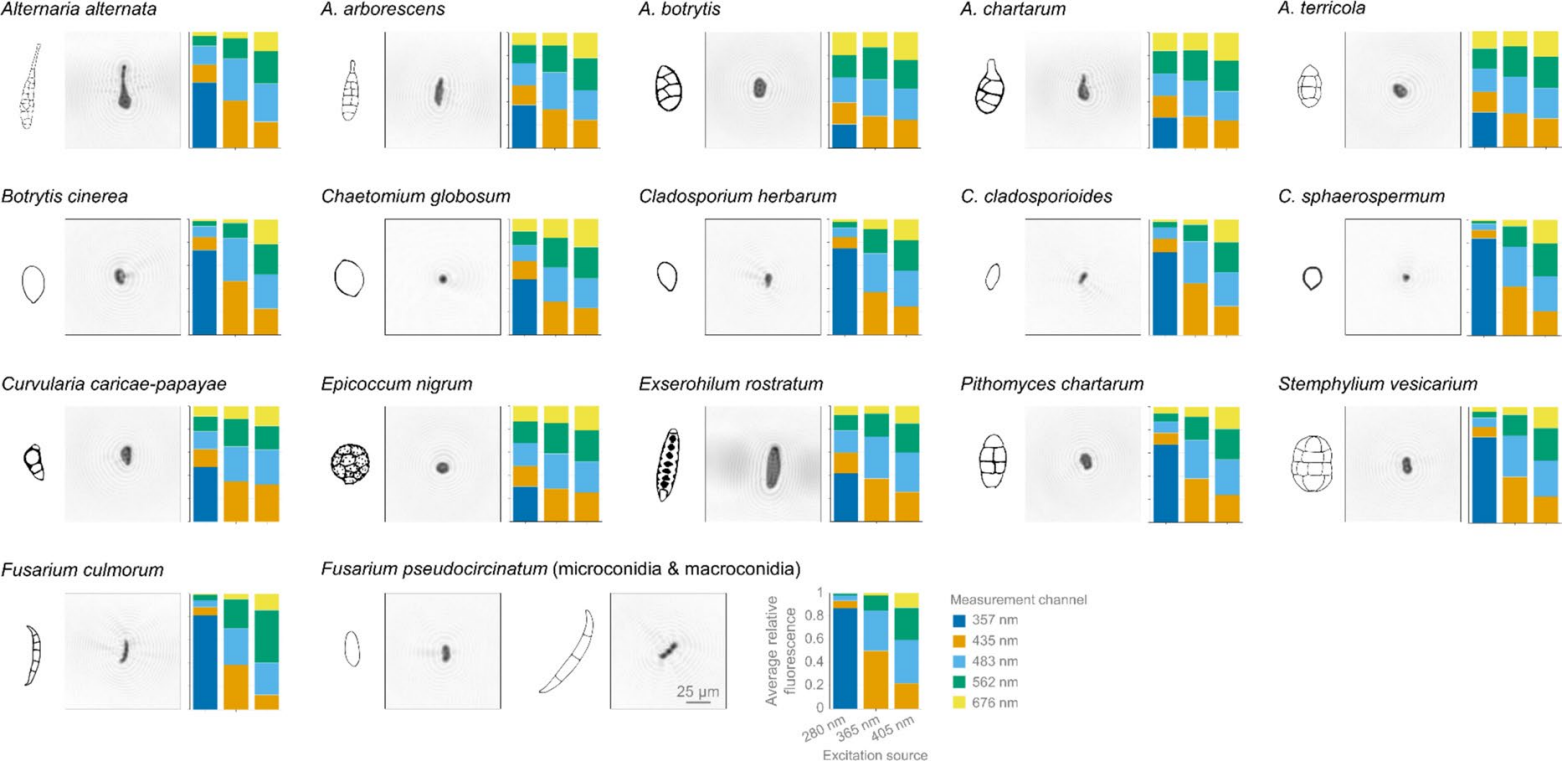
Holography images (120 × 120 µm) of representative single spore and datasets of average relative fluorescence (stacked barplots) recorded by the SwisensPoleno Jupiter, for 17 selected fungal species. Non-scaled representative drawings of spores adapted from Simmons EG, 2007 and the Atlas of Clinical Fungi (https://www.atlasclinicalfungi.org). Data are shown prior to any filtering for the creation of training datasets

For all measurements the instrument was operated at a trigger threshold low enough to measure particles with the size of at least 2 μm PSL, ensuring the detection of the smallest fungal spores used for this study. However, small particles may pose challenges to the holographic imaging, due to resolution constraints (0.595 μm per pixel), leading to measurements with just a single or no valid image captured. Only complete recordings of particles were used, even though valuable information might be present in one image, as well as the independently measured fluorescence spectrum (Supplementary Fig. 4). Holography images of representative single spores and the average relative fluorescence of the 17 tested fungal species are shown in Fig. 3, illustrating the recorded species-specific variability in morphology and fluorescence pattern. Median spore size for each species is shown in the Supplementary Table 1.

The aerosolization potential of various species was assessed, with scores ranging from 0 to 3 (Table 3) reflecting their propensity for aerosolization. Notably, *Alternaria botrytis*, *Chaetomium globosum*, *Curvularia caricae-papayae*, and *Epicoccum nigrum*, which exhibited the highest scores, indicating their ease of aerosolization, whereas *Alternaria chartarum* and *Cladosporium herbarum* scored the lowest. However, it is essential to recognize that these scores, indicative of particle flow stability, may not directly correlate with the recorded particle rate or the percentage of having two holographic images. For instance, despite receiving the lowest aerosolization score of 0, *Alternaria chartarum* demonstrated the highest percentage of valid holographic data. Indeed, all *Alternaria* species, with the exception of *A. alternata*, consistently yielded high percentages of two holographic data. Conversely, aerosolization of the three *Cladosporium* species were challenging, aligning with both lower particle rates and reduced percentage of valid holographic images (from 50 to 62%).

#### Exposure to the Plair Rapid-E+ 

**Fig. 4:**
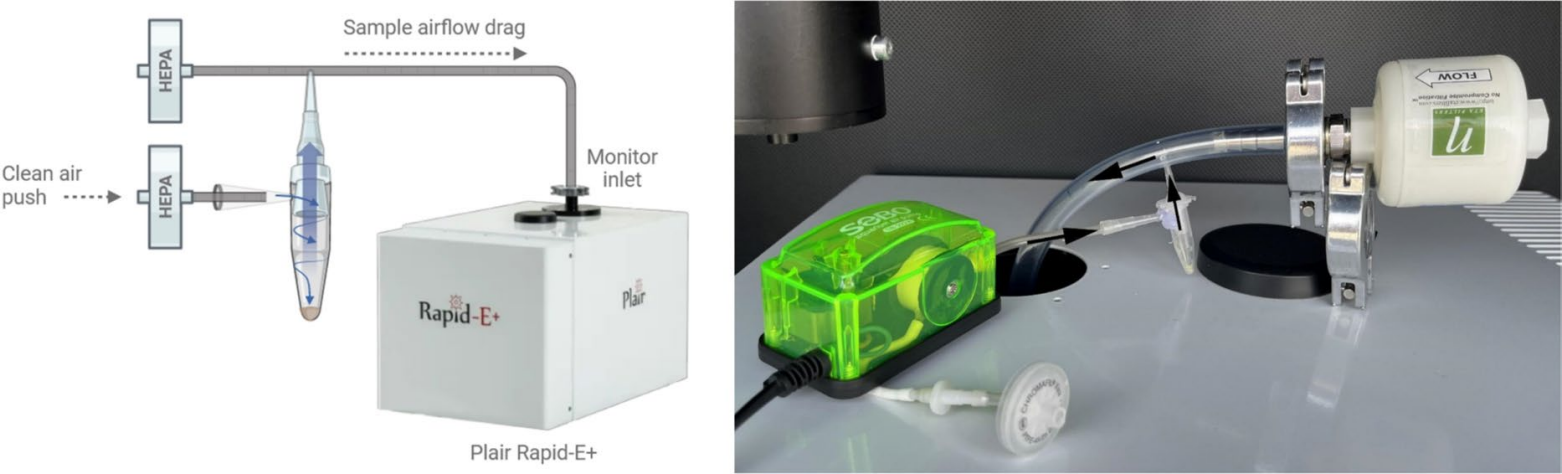
Experimental laboratory setup for the aerosolization of fungal spores and their detection by the Plair Rapid-E+. Arrows indicate the direction of air flows to and out of the cyclone collector vial

To address the absence of an atomizer compatible with the Plair Rapid-E+ (Plair SA), we devised a bespoke method based on analogous principles (Fig. 4). This approach was implemented using the cyclone collector detailed above. To aerosolize spores, the cyclone collector was connected to a sampling inlet via a plastic hose, with a low-resistance HEPA filter affixed to the free end. A low-volume pump was employed to draw air through the inlet, with the pump's inlet outfitted with a high-efficiency sterilization filter to prevent contaminants from entering the system and mixing with spores. The suction created by the Plair Rapid-E+ in conjunction with the airflow induced by the pump facilitated the cyclone's operation, selectively entraining a fraction of particles into the airstream directed towards the detection chamber. Particle release within the cyclone chamber was regulated by centrifugal forces, and agitation could be employed to increase it (either manual, via a vortex mixer, or using vibration from an electric toothbrush). All plastic components connecting the cyclone collector to the instrument were disposed of after each sample to avoid cross-contamination.

The Plair Rapid-E+ is an upgraded version of the earlier Plair Rapid-E, retaining the fundamental principles outlined in the previous version (Šaulienė et al., 2019) while introducing novel features (Sikoparija et al., 2024). Notably, measurements of scattering from 447 nm laser light are conducted at 14 angles, encompassing both parallel and perpendicular polarization planes. Fluorescence spectra are captured within the 350–530 nm range, with 32 measurements taken for each particle at a resolution of 12 nm. Additionally, fluorescence lifetimes are recorded across three spectral bands: 375–397, 415–450, and 467–487 nm. The device also incorporates supplementary scattering measurements from a 675 nm laser with a 4 × 4 pixel detector array. Operating at a sampling rate of 5 L of air per minute, the manufacturer asserts its capability to detect particles spanning sizes from 0.3 to 100 µm. Notably, the device offers three distinct detection modes, each characterized by varying sensitivity to particle size and fluorescence intensity. For the detection of fungal spores, the device was operated in the"middle"mode, facilitating comprehensive particle recording within the 1–100 µm size range. In a prior investigation, it was observed that the majority of fungal spores exhibit weak fluorescence upon excitation at 337 nm (Simović et al., 2023). Consequently, the Plair Rapid-E+ was configured to operate in the"middle"mode, wherein the gain of the spectrometer was increased by 28% and that of the fluorescence lifetime detectors by 10%, in comparison to the so-called “pollen” mode (Sikoparija et al., 2024). An inherent limitation of this mode is that the device is calibrated to detect small particles, larger than 1 µm in size, potentially resulting in the inclusion of additional debris and contaminants in the dataset.

The initial assessment of the collected datasets (Table 4) indicates that the efficiency of aerosolization was notably high, facilitating the collection of substantial quantities of fully measured particles within a brief timeframe. With the exception of *Cladosporium* spp.*, Curvularia caricae-papayae* and *Pithomyces chartarum*, the majority of spores from other fungal species exhibited fluorescence intensities with a maximum processed spectral signal exceeding 4000 units. This threshold, recommended by the instrument manufacturer, ensures that the fluorescence signal is sufficiently above the noise level to allow reliable analysis (Sikoparija et al., 2024). The limited fluorescence observed in *Cladosporium* spp., *Curvularia caricae-papayae*, and *Pithomyces chartarum* in our dataset may reflect a combination of biological and technical factors. Fluorescence emission varies significantly across fungal taxa, and as reported in previous studies (O’Connor et al., 2011; Saari et al., 2015), *Cladosporium* in particular is known to exhibit relatively weak intrinsic fluorescence, which may partially explain our findings. While further analysis is warranted to assess the signal quality, particularly regarding the fraction of particles where noise predominates, the acquired data proved adequate for conducting classification tests using convolutional neural network algorithms.

**Table 4:** Yield of aerosolization and exposure to the Plair Rapid-E+ for each fungal spore species, as evaluated by the percentage of exploitable fluorescence data

Species	Duration of aerosolization [min]	Total number of particles	Particles fluorescing above threshold [%]
*Alternaria* spp.	12	25,734	59
*Botrytis cinerea*	5	14,108	63
*Cladosporium* spp.	13	28,024	48
*Curvularia caricae-papayae*	13	14,784	44
*Epicoccum nigrum*	8	8871	62
*Exserohilum rostratum*	13	12,922	57
*Pithomyces chartarum*	8	14,021	50

*Alternaria* spp. include the mixed species *A. alternata*, *A. arborescens*, *A. botrytis*, *A. chartarum* and *A. terricola*. *Cladosporium* spp. include the mixed species *Cladosporium cladosporioides*, *Cladosporium herbarum* and *Cladosporium sphaerospermum*

## Fungal spore classification

Measurements of collected reference fungal spores by using the SwisensPoleno Jupiter and the Plair Rapid-E+ airflow cytometers were used to investigate the feasibility of classifying different spores. We evaluated the performance of each classification method using two metrics: taxon-level accuracy and global F1-score. Taxon-level accuracy is the proportion of correctly classified spore instances relative to the total number of analysed spores. The global F1-score, on the other hand, is the harmonic mean of precision and recall, calculated for each taxon, and then macro averaged.

### Identification by SwisensPoleno Jupiter

For the filtering of the captured datasets a simplistic approach was followed removing only particles with missing holographic images, particles with empty images, determined by a maximal grayscale intensity difference of the particles of 0.1, as well as particles far outside of the measurement channel centre (± 500 μm). There is no specific filtering applied to individual fungal species to prevent any bias introduction. However, this means there might be spore-agglomerates, hyphae or other non-spores particles present within the datasets. In contrast to Rapid-E+ data, no threshold for filtering out non-fluorescent particles was applied. However, restriction of allowed particle positions in the measurement channel, as well as removing empty holographic images reduces the possibility of having data solely representing noise.

Two classification models were trained for the SwisensPoleno. First, using holographic images only and then using both holography and relative fluorescence spectrum measurements. The holographic model utilizes a modified EfficientNet B0 architecture (Tan & Le, 2020) that can leverage the information from the two holographic images. Each image is first encoded by the EfficientNet B0 architecture without its final classification layer. Subsequently, the extracted features from both images are combined via a series of feedforward layers following a layer normalization step. This combined representation is then fed into the final classification layer. For models incorporating fluorescence data, an additional input branch is introduced. This branch utilizes a feedforward layer to encode the spectral information. The encoded fluorescence data is then concatenated with the combined embedding from the other images before feeding into the final classification layer. In both cases, the entire network is trained end-to-end from scratch.

For both models, 80% of the data was used for training, 10% for hyperparameter tuning and the remaining 10% to test the classification performance. Training, validation and test datasets are exactly the same for both models.

**Fig. 5:**
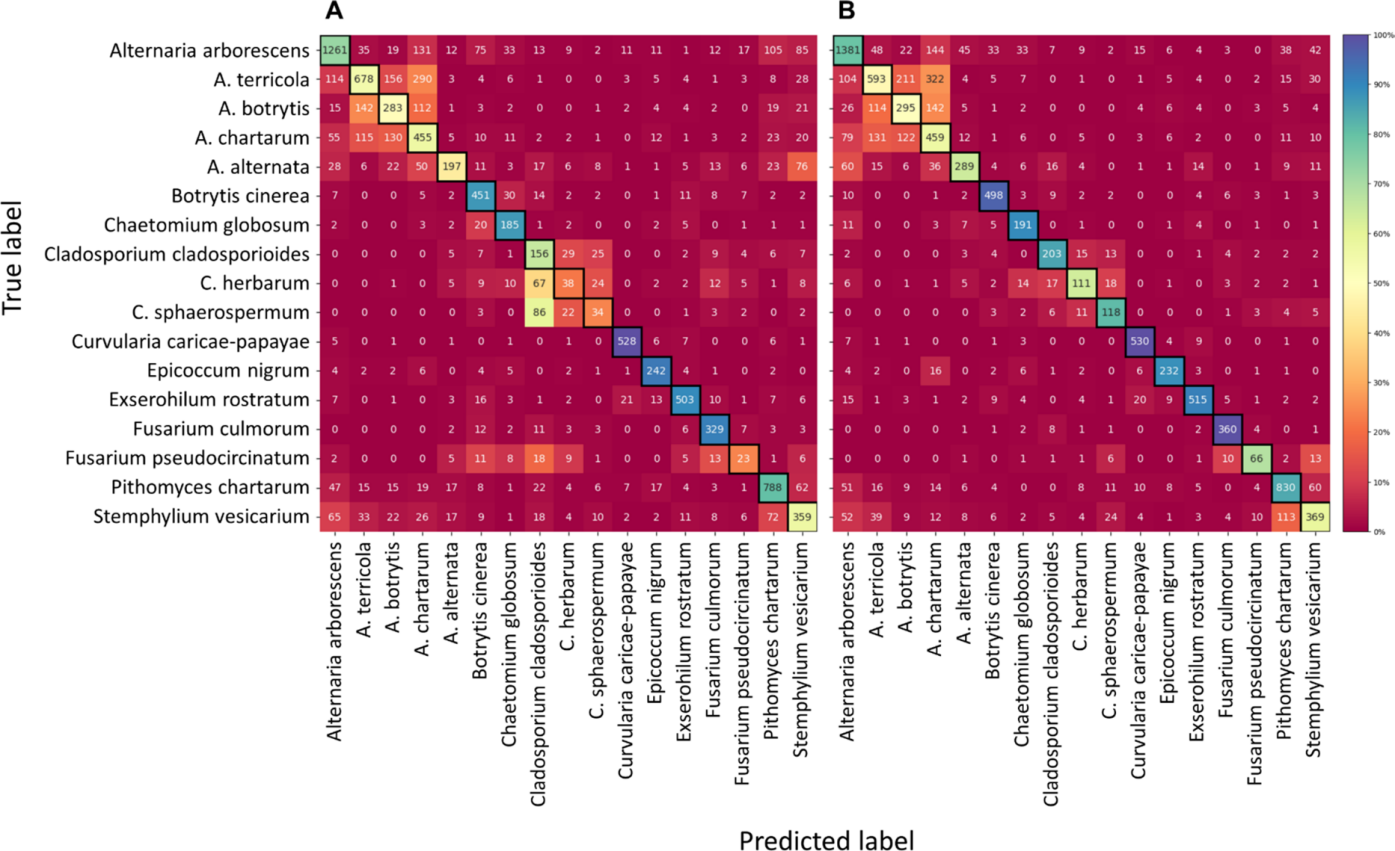
Confusion matrices of the recognition performance of the convolutional neural networks trained on (**A**) holography imaging data or (**B**) combined holography imaging and fluorescence data from 17 fungal spore taxa recorded by the SwisensPoleno Jupiter. Percentage of accurately predicted and misclassified test particles is presented as a heat map

The classification results of the 17 selected fungal species (Fig. 5; Supplementary Table 2), showed a significant improvement between the holography-only and the holography-fluorescence models. The first reached an F1-score of 0.60 and the second of 0.72. This enhancement underscores the complementary nature of holographic and fluorescence modalities in discerning subtle differences among fungal spore species. This is particularly the case among the three analysed *Cladosporium* species for which accuracy increased from 62.2 to 80.9% for *C. cladosporioides*, from 20.7 to 60.3% for *C. herbarum*, and from 22.2 to 77.1% for *C. sphaerospermum*. Notably, the accuracy observed for *Fusarium pseudocircinatum* strongly increased from 22.5 to 64.7%, which is probably due to the morphologically non homogenous mix of macroconidia and microconidia.

The SwisensPoleno Jupiter demonstrated robust performance if classes are aggregated by genus. When employing solely holographic data for classification, the F1-score reached 0.77, indicative of the best system efficacy in distinguishing between different fungal genera among the two monitors tested in this study. Upon integration of fluorescence data alongside holographic imagery, the classification accuracy could even be further improved, yielding an elevated F1-score of 0.83. In particular, *Curvularia caricae-papayae* exhibited the highest accuracy at 95.2%, followed closely by *Alternaria* spp. (grouping *A. alternata*, *A. arborescens*, *A. botrytis*, *A. chartarum* and *A. terricola*) and *Botrytis cinerea* at 91.5%.

### Identification by the Plair Rapid-E+ 

In this study, we employed an analytical methodology derived from the Plair Rapid-E framework (Tešendić et al., 2022), integrating multiple measurement modalities. These modalities encompassed parallel and perpendicular polarization scattering, infrared scattering, fluorescence spectrum, and fluorescence lifetime data. To ensure the accuracy of our measurements, we applied established filtering techniques, excluding data points where the maximum intensity of the spectrum fell below 4000 units, thereby minimizing the inclusion of erroneous noise artefacts. Our preprocessing pipeline prioritized simplicity to bolster the robustness of our classification model. We removed fluorescence measurements at the 7 th (about 450 nm) and 8 th (about 462 nm) detectors, which were susceptible to interference from the light of the scattering laser. Additionally, we aligned the fluorescence lifetime measurements so that the signal starts at the first maximum. To enhance data quality, the fluorescence spectrum and all three scattering images were smoothed using a Savitzky–Golay filter. Finally, we transformed both the fluorescence spectrum and the fluorescence lifetime modalities into image-like formats and then normalized them into a 0–1 range to facilitate uniform handling by the convolutional neural networks.

For classification of selected fungal spores, we implemented a ResNet network, which showed superior performance in a previous study with the Plair Rapid-E device (Matavulj et al., 2023). The architecture employed in ResNet features that are termed as"shortcut connections". When an input, represented as x, passes through the convolutional layers of the network, it undergoes a transformation denoted by the function F. This function F(x) serves to highlight key features such as edges and shapes, which are critical to the accurate representation of the original image. The shortcut connection introduces x to the F(x), so the output x + F(x) represents a slightly altered or refined version of the original input x. We utilized a variation of an 18-layer ResNet model, where we implemented certain layers of ResNet. Specifically, we implemented a 4-block-layer ResNet for fluorescence lifetime, a 3-block-layer for scattering images, a 5-block-layer net for fluorescence spectrum, and a 2-block-layer ResNet for infrared. The block-layers contained 3 convolutional layers (except for the first block-layer, which comprised 4 convolutional layers). To handle fluorescence lifetime and spectrum data, the first convolutional layer was customized to accept a monochrome image, configured with a kernel size of 5 × 5, a padding of 2 × 2, and without any stride to maintain the original spatial dimensions. To address our classification task with its defined number of classes, we adjusted the final fully-connected layer accordingly. The classification model was trained on 80% of the reference dataset, 10% was used for model validation during training to prevent overfitting, and 10% was used to test the classification performance.

**Fig. 6:**
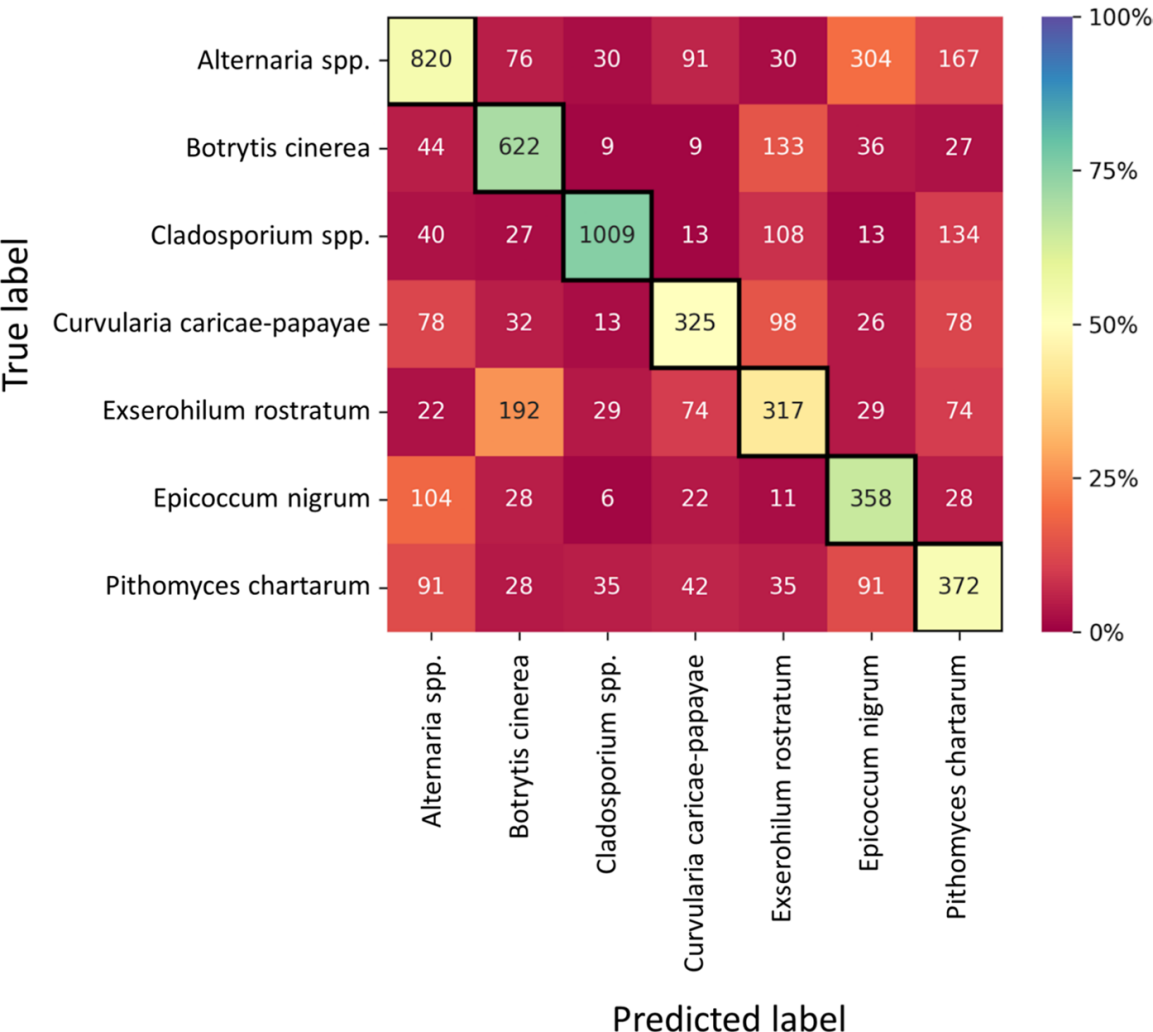
Confusion matrix of the recognition performance of the convolutional neural networks trained on fluorescence data from seven fungal spore genera recorded by the Plair Rapid-E+. *Alternaria* spp. include the mixed species *A. alternata*, *A. arborescens*, *A. botrytis*, *A. chartarum* and *A. terricola*. *Cladosporium* spp. include the mixed species *Cladosporium cladosporioides*, *Cladosporium herbarum* and *Cladosporium sphaerospermum*. Percentage of accurately predicted and misclassified test particles is presented as a heat map

The classification performance of selected fungal spores measured with the Plair Rapid-E+ device yielded a macro average F1-score exceeding 0.61 (Fig. 6 and Supplementary Table 3). Notably, the model demonstrated best accuracy in identifying *Curvularia caricae-papayae* (accuracy 95.1%) and *Cladosporium* spp. (accuracy 92.7%), followed by *Botrytis cinerea* (accuracy 89.9%) and *Epicoccum nigrum* (accuracy 88.9%). Both *Botrytis cinerea* and *Epicoccum nigrum* had the most intense fluorescence after excitation at 337 nm and *Cladosporium* spp. was distinctively smaller than other tested spores. The analysis confirmed the potential of Plair Rapid-E+ measurements in the “middle” mode to discriminate between some of the most prevalent airborne fungal spores. However, since it is known that laser-induced data are prone to noise resulting from laser and detector sensibility (Huffman et al., 2020; Könemann et al., 2019; Robinson et al., 2017), more work needs to be done on cleaning training datasets to further improve the recognition performance.

## Discussion and conclusion

The study elucidates effective methodologies for fungal spore harvesting and aerosolization, crucial steps in the creation of training datasets for automated monitoring systems. It emphasizes the importance of representative fungal strains, controlled sporulation, and harvesting of individual spores. Dry extraction of in vitro fungi cultures on agar medium using a cyclone collector proves to be an optimal approach for maintaining spore integrity, minimizing hyphal contamination, and allowing to produce a stock of reference spore material that can easily be stored and shared.

Furthermore, several recommendations emerged from this protocol of spore production:

1. *Careful strain selection* The selection of specific fungal strains is paramount. We have shown that it may significantly affect morphology-based identification accuracy, in particular for large complexes like *Alternaria* and *Cladosporium*. The presence of mixed macroconidia and microconidia, as observed in *Fusarium*, is also a point that should be taken into account, as it may alter identification efficiency.
2. *Anticipate phenotype variability* Relatively variable results from a same fungal culture protocol is inevitable across different laboratories. Factors such as strain fitness, growth medium, and duration can influence outcomes. It is essential to allow sufficient growth time to ensure a diverse range of spore maturity and enhance the representativeness of the reference sample. However, for some strains, excessively long culture periods may lead to the growth of secondary mycelium, thus reducing the sporulation yield on the surface of Petri dishes. This can though be mitigated by an increased amount of dishes.
3. *Dry spores from dry cultures* While humidity control during the culture growth period may not be critical, it is recommended to dry Petri dishes just before the harvesting process in order to facilitate efficient spore collection. Cultures exhibiting high humidity levels, often indicated by water exudates within the mycelium, can impede the gentle harvest of spores, which is essential for minimizing the inadvertent sampling of hyphae. In particular, the spore dispersal strategy of some fungi (e.g., *Acremonium* spp., *Stachybotrys chartarum*, *Fusarium* spp., some myxomycetes, etc.) relies on the production of droplets or wet false heads at the apex of conidiophores (Magyar et al., 2016), which might be incompatible with the proposed protocol or would require some adaptation in regard to humidity level. Additionally, ambient humidity may influence the fluorescence intensity of biogenic secondary organic aerosols (Zhang et al., 2021), potentially posing a caveat to our proposed method for aerosolizing dry fungal spores.

There is considerable potential for optimizing the method presented in this study. However, before assessing the classification performance in an operational setup against standard measurements such as EN 16868, several aspects pertaining to spore’s variability should be addressed by the scientific community in aerobiology.

While the variability in spore morphology within a single culture is generally well-documented for reference fungal strains, there is limited understanding of how this cultured variability correlates with the possible range of spore variability in natural environments. Single spores might not constitute the sole form suspended in the atmosphere. Moreover, most studies have only focussed on fresh bioaerosols for setting up datasets for machine learning. After long term transportation in ambient air, fungal spores might undergo changes in morphology and chemical properties due to ageing and interactions with atmospheric components such as pollutants, humidity and UV radiation. This represents a key research gap. Compounding this issue, spores cultivated in artificial nutrient media can exhibit diverse maturation states. For instance, immature spores of *Alternaria* may appear hyaline, ovoid, and one or two-celled, significantly different from their mature, multicellular forms. Such heterogeneity complicates the development of standardized and representative reference datasets for training reliable classification models.

Moreover, the cleaning procedures employed will significantly influence the nature of the classification model and, consequently, its performance when compared to validation datasets like standard aerobiological measurements. For example, if standard measurements encompass both spores and hyphae under a specific spore type, cleaning protocols should refrain from discarding hyphae. Furthermore, for spores suspended in the atmosphere as aggregates (e.g., chains of *Cladosporium* conidia), retaining such formations in the training dataset becomes imperative. However, it is essential to note that when analysed under an upright microscope, a chain of three spores should be treated as a single particle rather than three separate ones, to ensure methodological comparability.

Finally, exploring the chemical characteristics of spore cell walls from such reference material would be important, especially in cases where fluorescence measurements are utilized for identification. As previous observations showing that sporulation age significantly affects signal response (Kanaani et al., 2007; Saari et al., 2015), investigating chemical specificities given variable culture conditions would provide insights into the factors influencing algorithmic confusion and aid in the development of more accurate and reliable automated identification systems.

In all, these considerations are crucial for the accurate interpretation and comparison of data obtained through different methodologies and instruments in the field of aerobiology. Moving forward, efforts should focus on expanding reference datasets to encompass a broader spectrum of fungal diversity found in the atmosphere. This will facilitate more accurate and comprehensive automated identification of fungal spores, ultimately enhancing our understanding of bioaerosol dynamics and their impact on human and plant health.

## Supplementary Information

Below is the link to the electronic supplementary material.

Supplementary file1 (DOCX 19522 KB)

## Data Availability

The data that support the findings of this study are available from the corresponding author (NB) upon reasonable request.

## Footnotes

1 https://bccm.belspo.be/catalogues/ihem-catalogue-search.

2 https://bccm.belspo.be/catalogues/ihem-media-details?CultureMediaID=905.

3 https://bccm.belspo.be/catalogues/ihem-media-details?CultureMediaID=908.

4 https://bccm.belspo.be/catalogues/ihem-media-details?CultureMediaID=902.

## References

[CR1] Ajmal, M., Hussain, A., Ali, A., Chen, H., & Lin, H. (2022). Strategies for controlling the sporulation in *Fusarium* spp. *JoF,**9*, 10. 10.3390/jof9010010 36675831 10.3390/jof9010010 PMC9861637

[CR2] Anees-Hill, S., Douglas, P., Pashley, C. H., Hansell, A., & Marczylo, E. L. (2022). A systematic review of outdoor airborne fungal spore seasonality across Europe and the implications for health. *Science of the Total Environment,**818*, Article 151716. 10.1016/j.scitotenv.2021.151716 34800445 10.1016/j.scitotenv.2021.151716 PMC8919338

[CR3] Atlas, R. M., & Parks, L. S. (1993). *Handbook of microbiological media* (pp. 1079–1079). CRC.

[CR4] Banchi, E., Ametrano, C. G., Tordoni, E., Stankovic, D., Ongaro, S., & ARPA working group, Tretiach, M., Pallavicini, A., Muggia, L.,. (2020). Environmental DNA assessment of airborne plant and fungal seasonal diversity. *Science of the Total Environment,**738*, Article 140249. 10.1016/j.scitotenv.2020.140249 32806340 10.1016/j.scitotenv.2020.140249

[CR5] BCCM/IHEM (2015). YouTube, uploaded by BCCM/IHEM, “BCCM/IHEM freeze-dried ampoules: instructions for use”, https://www.youtube.com/watch?v=9nKyMwOxTTA

[CR6] Bensch, K., Groenewald, J. Z., Braun, U., Dijksterhuis, J., De Jesús Yáñez-Morales, M., & Crous, P. W. (2015). Common but different: The expanding realm of Cladosporium. *Studies in Mycology,**82*, 23–74. 10.1016/j.simyco.2015.10.001 26955200 10.1016/j.simyco.2015.10.001 PMC4774271

[CR7] Buters, J., Clot, B., Galán, C., Gehrig, R., Gilge, S., Hentges, F., O’Connor, D., Sikoparija, B., Skjoth, C., Tummon, F., Adams-Groom, B., Antunes, C. M., Bruffaerts, N., Çelenk, S., Crouzy, B., Guillaud, G., Hajkova, L., Seliger, A. K., Oliver, G., … Stjepanovic, B. (2022). Automatic detection of airborne pollen: An overview. *Aerobiologia*. 10.1007/s10453-022-09750-x

[CR8] Crawford, I., Bower, K., Topping, D., Di Piazza, S., Massabò, D., Vernocchi, V., & Gallagher, M. (2023). Towards a UK airborne bioaerosol climatology: Real-time monitoring strategies for high time resolution bioaerosol classification and quantification. *Atmosphere,**14*(8), 1214.

[CR9] Després, V. R., Huffman, J. A., Burrows, S. M., Hoose, C., Safatov, A. S., Buryak, G., Fröhlich-Nowoisky, J., Elbert, W., Andreae, M. O., Pöschl, U., & Jaenicke, R. (2012). Primary biological aerosol particles in the atmosphere: A review. *Tellus b: Chemical and Physical Meteorology,**64*, 15598. 10.3402/tellusb.v64i0.15598

[CR10] Drew Smith, J., Crawley, W. E., & Lees, F. T. (1961). Collection and concentration of spores of *Pithomyces chartarum* from herbage and harvesting from ryecorn cultures. *New Zealand Journal of Agricultural Research,**4*, 725–733. 10.1080/00288233.1961.10431629

[CR11] EN 16868, (2019). ‘Ambient air - Sampling and analysis of airborne pollen grains and fungal spores for networks related to allergy - Volumetric Hirst method’, Cen.

[CR12] Erb, S., Berne, A., Burgdorfer, N., Clot, B., Graber, M.-J., Lieberherr, G., Sallin, C., Tummon, F., & Crouzy, B. (2023). Automatic real-time monitoring of fungal spores: The case of Alternaria spp. *Aerobiologia*. 10.1007/s10453-023-09780-z 10.1007/s10453-023-09780-z PMC11096102 38766603

[CR13] Fröhlich-Nowoisky, J., Kampf, C. J., Weber, B., Huffman, J. A., Pöhlker, C., Andreae, M. O., Lang-Yona, N., Burrows, S. M., Gunthe, S. S., Elbert, W., Su, H., Hoor, P., Thines, E., Hoffmann, T., Després, V. R., & Pöschl, U. (2016). Bioaerosols in the earth system: Climate, health, and ecosystem interactions. *Atmospheric Research,**182*, 346–376. 10.1016/j.atmosres.2016.07.018

[CR14] Galán, C., Smith, M., Damialis, A., Frenguelli, G., Gehrig, R., Grinn-Gofroń, A., Kasprzyk, I., Magyar, D., Oteros, J., Šaulienė, I., Thibaudon, M., Sikoparija, B., EAS QC Working Group. (2021). Airborne fungal spore monitoring: between analyst proficiency testing. *Aerobiologia,**37*, 351–361. 10.1007/s10453-021-09698-4

[CR15] González-Alonso, M., Boldeanu, M., Koritnik, T., Gonçalves, J., Belzner, L., Stemmler, T., Gebauer, R., Grewling, Ł, Tummon, F., Maya-Manzano, J. M., Ariño, A. H., Schmidt-Weber, C., & Buters, J. (2023). Alternaria spore exposure in Bavaria, Germany, measured using artificial intelligence algorithms in a network of BAA500 automatic pollen monitors. *Science of the Total Environment,**861*, Article 160180. 10.1016/j.scitotenv.2022.160180 36403848 10.1016/j.scitotenv.2022.160180

[CR16] Healy, D. A., O’Connor, D. J., Burke, A. M., & Sodeau, J. R. (2012). A laboratory assessment of the Waveband Integrated Bioaerosol Sensor (WIBS-4) using individual samples of pollen and fungal spore material. *Atmospheric Environment,**60*, 534–543. 10.1016/j.atmosenv.2012.06.052

[CR50] Hill, S. C., Pan, Y. L., Williamson, C., Santarpia, J. L. & Hill, H. H., (2013). Fluorescence of bioaerosols: mathematical model including primary fluorescing and absorbing molecules in bacteria. *Optics Express*, *21*(19), 22285–22313 24104120 10.1364/OE.21.022285

[CR17] Hoekstra, F. (1995). Collecting pollen for genetic resources conservation. In: L. Guarino, V. Ramanatha Rao, R. Reid, (eds.), *Collecting Plant Genetic Diversity: Technical Guidelines*, (pp. 527–550) Wallingford, UK.

[CR18] Huffman, J. A., Sinha, B., Garland, R., Snee-Pollmann, A., Gunthe, S., Artaxo, P., et al. (2012). Size distributions and temporal variations of biological aerosol particles in the Amazon rainforest characterized by microscopy and real-time UV-APS fluorescence techniques during AMAZE-08. *Atmospheric Chemistry and Physics,**12*, 11997–12019. 10.5194/acp-12-11997-2012

[CR19] Huffman, J. A., Perring, A. E., Savage, N. J., Clot, B., Crouzy, B., Tummon, F., Shoshanim, O., Damit, B., Schneider, J., Sivaprakasam, V., Zawadowicz, M. A., Crawford, I., Gallagher, M., Topping, D., Doughty, D. C., Hill, S. C., & Pan, Y. (2020). Real-time sensing of bioaerosols: Review and current perspectives. *Aerosol Science and Technology,**54*, 465–495. 10.1080/02786826.2019.1664724

[CR20] Jacques, S., Lenzo, L., Stevens, K., Lawrence, J., & Tan, K.-C. (2021). An optimized sporulation method for the wheat fungal pathogen Pyrenophora tritici-repentis. *Plant Methods,**17*, 52. 10.1186/s13007-021-00751-4 34011363 10.1186/s13007-021-00751-4 PMC8136220

[CR21] Kanaani, H., Hargreaves, M., Ristovski, Z., & Morawska, L. (2007). Performance assessment of UVAPS: Influence of fungal spore age and air exposure. *Journal of Aerosol Science,**38*, 83–96. 10.1016/j.jaerosci.2006.11.003

[CR22] Könemann, T., Savage, N., Klimach, T., Walter, D., Fröhlich-Nowoisky, J., Su, H., Pöschl, U., Huffman, J. A., & Pöhlker, C. (2019). Spectral Intensity Bioaerosol Sensor (SIBS): An instrument for spectrally resolved fluorescence detection of single particles in real time. *Atmospheric Measurement Techniques,**12*, 1337–1363. 10.5194/amt-12-1337-2019

[CR23] Li, X., & Fu, H. (2020). Fungal spore aerosolization at different positions of a growing colony blown by airflow. *Aerosol and Air Quality Research,**20*, 2826–2833. 10.4209/aaqr.2020.09.0565

[CR24] Lieberherr, G., Auderset, K., Calpini, B., Clot, B., Crouzy, B., Gysel-Beer, M., Konzelmann, T., Manzano, J., Mihajlovic, A., Moallemi, A., O’Connor, D., Sikoparija, B., Sauvageat, E., Tummon, F., & Vasilatou, K. (2021). Assessment of real-time bioaerosol particle counters using reference chamber experiments. *Atmospheric Measurement Techniques,**14*, 7693–7706. 10.5194/amt-14-7693-2021

[CR25] Magyar, D., Vass, M., & Li, D. W. (2016). Dispersal Strategies of Microfungi. In D. W. Li (Ed.), *Biology of microfungi. Fungal biology. *Springer. 10.1007/978-3-319-29137-6_14

[CR26] Markey, E., Clancy, J. H., Martínez-Bracero, M., Sarda-Estève, R., Baisnée, D., McGillicuddy, E. J., Sewell, G., Skjøth, C., & O’Connor, D. J. (2024). Spectroscopic detection of bioaerosols with the wibs-4+: Anthropogenic and meteorological impacts. *Science of the Total Environment,**943*, 173649. 38852865 10.1016/j.scitotenv.2024.173649

[CR27] Matavulj, P., Panić, M., Šikoparija, B., Tešendić, D., Radovanović, M., & Brdar, S. (2023). Advanced CNN architectures for pollen classification: Design and comprehensive evaluation. *Applied Artificial Intelligence,**37*, 2157593. 10.1080/08839514.2022.2157593

[CR28] Maya-Manzano, J. M., Tummon, F., Abt, R., Allan, N., Bunderson, L., Clot, B., Crouzy, B., Daunys, G., Erb, S., Gonzalez-Alonso, M., Graf, E., Grewling, Ł, Haus, J., Kadantsev, E., Kawashima, S., Martinez-Bracero, M., Matavulj, P., Mills, S., Niederberger, E., … Buters, J. (2023). Towards European automatic bioaerosol monitoring: Comparison of 9 automatic pollen observational instruments with classic Hirst-type traps. *Science of the Total Environment,**866*, Article 161220. 10.1016/j.scitotenv.2022.161220 36584954 10.1016/j.scitotenv.2022.161220

[CR29] O’Connor, D. J., Iacopino, D., Healy, D. A., O’Sullivan, D., & Sodeau, J. R. (2011). The intrinsic fluorescence spectra of selected pollen and fungal spores. *Atmospheric Environment,**45*(35), 6451–6458.

[CR30] Pogner, C. E., Graf, E., Niederberger, E., & Gorfer, M. (2024). What do spore particles look like - use of real-time measurements and holography imaging to view spore particles from four bioaerosol generators. *Aerosol Science and Technology,**58*(7), 779–795. 10.1080/02786826.2024.2338544

[CR31] Pöhlker, C., Huffman, J. A., & Pöschl, U. (2011). Autofluorescence of atmospheric bioaerosols–fluorescent biomolecules and potential interferences. *Atmospheric Measurement Techniques Discussions,**4*(5), 5857–5933.

[CR32] Robinson, E. S., Gao, R.-S., Schwarz, J. P., Fahey, D. W., & Perring, A. E. (2017). Fluorescence calibration method for single-particle aerosol fluorescence instruments. *Atmospheric Measurement Techniques Discussions,**10*, 1755–1768. 10.5194/amt-10-1755-2017

[CR33] Saari, S., Mensah-Attipoe, J., Reponen, T., Veijalainen, A. M., Salmela, A., Pasanen, P., & Keskinen, J. (2015). Effects of fungal species, cultivation time, growth substrate, and air exposure velocity on the fluorescence properties of airborne fungal spores. *Indoor Air,**25*(6), 653–661. 25292152 10.1111/ina.12166

[CR34] Šaulienė, I., Šukienė, L., Daunys, G., Valiulis, G., Vaitkevičius, L., Matavulj, P., Brdar, S., Panic, M., Sikoparija, B., Clot, B., Crouzy, B., & Sofiev, M. (2019). Automatic pollen recognition with the Rapid-E particle counter: The first-level procedure, experience and next steps. *Atmos. Meas. Tech.,**12*, 3435–3452. 10.5194/amt-12-3435-2019

[CR35] Sauvageat, E., Zeder, Y., Auderset, K., Calpini, B., Clot, B., Crouzy, B., Konzelmann, T., Lieberherr, G., Tummon, F., & Vasilatou, K. (2020). Real-time pollen monitoring using digital holography. *Atmos. Meas. Tech.,**13*, 1539–1550. 10.5194/amt-13-1539-2020

[CR36] Scheermeyer, E., & Agranovski, I. E. (2009). Design and evaluation of a new device for fungal spore aerosolization for laboratory applications. *Journal of Aerosol Science,**40*(10), 879–889.

[CR37] Senanayake, I. (2020). Morphological approaches in studying fungi: Collection, examination, isolation, sporulation and preservation. *Mycosphere,**11*, 2678–2754. 10.5943/mycosphere/11/1/20

[CR38] Sikoparija, B., Matavulj, P., Simovic, I., Radisic, P., Brdar, S., Minic, V., Tesendic, D., Kadantsev, E., Palamarchuk, J., & Sofiev, M. (2024). Classification accuracy and compatibility across devices of a new Rapid-E+ flow cytometer. Atmospheric Measurement Techniques. 10.5194/egusphere-2024-187

[CR39] Simmons, E. G. (2007). Alternaria: An Identification Manual. CBS Fungal Biodiversity Centre, Utrecht, NL.

[CR40] Simović, I., Matavulj, P., & Šikoparija, B. (2023). Manual and automatic quantification of airborne fungal spores during wheat harvest period. *Aerobiologia,**39*, 227–239. 10.1007/s10453-023-09788-5

[CR41] Su, Y.-Y., Qi, Y.-L., & Cai, L. (2012). Induction of sporulation in plant pathogenic fungi. *Mycology,**3*, 195–200. 10.1080/21501203.2012.719042

[CR42] Tan, M., Le, Q. V. (2019). EfficientNet: Rethinking Model Scaling for Convolutional Neural Networks. 10.48550/ARXIV.1905.11946

[CR43] Tešendić, D., Boberić Krstićev, D., Matavulj, P., Brdar, S., Panić, M., Minić, V., & Šikoparija, B. (2022). RealForAll: Real-time system for automatic detection of airborne pollen. *Enterprise Information Systems,**16*, 1793391. 10.1080/17517575.2020.1793391

[CR44] Tordoni, E., Ametrano, C. G., Banchi, E., Ongaro, S., Pallavicini, A., Bacaro, G., & Muggia, L. (2021). Integrated eDNA metabarcoding and morphological analyses assess spatio-temporal patterns of airborne fungal spores. *Ecological Indicators,**121*, Article 107032. 10.1016/j.ecolind.2020.107032

[CR45] Tummon, F., Arboledas, L. A., Bonini, M., Guinot, B., Hicke, M., Jacob, C., Kendrovski, V., McCairns, W., Petermann, E., Peuch, V., Pfaar, O., Sicard, M., Sikoparija, B., & Clot, B. (2021). The need for Pan-European automatic pollen and fungal spore monitoring: A stakeholder workshop position paper. *Clinical & Translational All,**11*, Article e12015. 10.1002/clt2.12015 10.1002/clt2.12015 PMC8120382 33934521

[CR46] Tummon, F., Bruffaerts, N., Celenk, S., Choël, M., Clot, B., Crouzy, B., Galán, C., Gilge, S., Hajkova, L., Mokin, V., O’Connor, D., Rodinkova, V., Sauliene, I., Sikoparija, B., Sofiev, M., Sozinova, O., Tesendic, D., & Vasilatou, K. (2022). Towards standardization of automatic pollen and fungal spore monitoring: Best practices and guidelines. *Aerobiologia*. 10.1007/s10453-022-09755-6

[CR47] Tummon, F., Adams-Groom, B., Antunes, C. M., Bruffaerts, N., Buters, J., Cariñanos, P., Celenk, S., Choël, M., Clot, B., Cristofori, A., Crouzy, B., Damialis, A., Fernández, A. R., González, D. F., Galán, C., Gedda, B., Gehrig, R., Gonzalez-Alonso, M., Gottardini, E., … de Weger, L. A. (2024). The role of automatic pollen and fungal spore monitoring across major end-user domains. *Aerobiologia*. 10.1007/s10453-024-09820-2

[CR48] Wounderberg, J. H. C., Seidl, M. F., Groenewald, J. Z., de Vries, M., Stielow, J. B., Thomma, B. P. H. J., & Crous, P. W. (2015). *Alternaria* section *Alternaria*: Species, formae speciales or pathotypes? *Studies in Mycology,**82*, 1–21. 10.1016/j.simyco.2015.07.001 26951037 10.1016/j.simyco.2015.07.001 PMC4774270

[CR49] Zhang, M., Su, H., Li, G., Kuhn, U., Li, S., Klimach, T., Hoffmann, T., Fu, P., Pöschl, U., & Cheng, Y. (2021). High-resolution fluorescence spectra of airborne biogenic secondary organic aerosols: Comparisons to primary biological aerosol particles and implications for single-particle measurements. *Environmental Science & Technology,**55*(24), 16747–16756. 10.1021/acs.est.1c02536 34699200 10.1021/acs.est.1c02536 PMC8697557

