# Supplementary material for: Advancing automated identification of airborne fungal spores: guidelines for cultivation and reference dataset creation
Source: Aerobiologia (Bologna). 2025 Jun 2;41(2):505–25. doi: 10.1007/s10453-025-09864-y (PMC12176942; doi:10.1007/s10453-025-09864-y)
Supplement: Supplementary file 1 — Supplementary file1 (DOCX 19522 KB) [file 10453_2025_9864_MOESM1_ESM.docx]

**Supplementary Table 1**

Size of spores measured from the holography images of the SwisensPoleno Jupiter, as compared to the expected spore length described in the Atlas of Clinical Fungi ([https://www.atlasclinicalfungi.org](https://www.atlasclinicalfungi.org/)). The major axis is an average calculated from the median major axis *holo0* and the median major axis *holo1*. The equivalent diameter (D) was calculated from the average area (A) between the median area *holo0* and the median area *holo1* as following: D=2*sqrt(A/π).

| **Species** | **Expected length [µm]** | **Measured major axis [µm]** | **Measured equivalent diameter [µm]** |
| --- | --- | --- | --- |
| *Alternaria* *alternata* | 22-95 | 25.3 | 17.7 |
| *Alternaria arborescens* | 23-56 | 16.0 | 12.3 |
| *Alternaria botrytis* | 19-25 | 15.2 | 13.9 |
| *Alternaria chartarum* | 18-38 | 13.6 | 12.3 |
| *Alternaria terricola* | 15-32 | 13.9 | 12.7 |
| *Botrytis cinerea* | 8-14 | 12.3 | 9.7 |
| *Chaetomium globosum* | 9-12 | 9.8 | 8.3 |
| *Cladosporium cladosporioides* | 2-6 | 17.3 | 13.4 |
| *Cladosporium herbarum* | 4-9 | 18.9 | 13.9 |
| *Cladosporium sphaerospermum* | 3-5 | 18.9 | 14.2 |
| *Curvularia caricae-papayae* | 21-31 | 18.3 | 13.6 |
| *Epicoccum nigrum* | 9-11 | 11.7 | 10.4 |
| *Exserohilum rostratum* | 30-128 | 33.3 | 19.6 |
| *Fusarium culmorum* | 30-45 (macroconidia) | 20.6 | 11.5 |
| *Fusarium pseudocircinatum* | 23-54 (macroconidia)  5-12  (microconidia) | 12.9 | 10.0 |
| *Pithomyces* *chartarum* | 10-20 | 16.5 | 13.3 |
| *Stemphylium vesicarium* | 15-30 | 19.4 | 14.6 |

**Supplementary Table 2**

Precision, recall, accuracy and F1-score values from classification with the SwisensPoleno Jupiter system, based on holography imaging or combined holography imaging and fluorescence data. Performance was calculated with the same classifier for genera by combining species labels.

|  | **Holography** | | | | **Holography and Fluorescence** | | | |
| --- | --- | --- | --- | --- | --- | --- | --- | --- |
| **Species** | **Precision** | **Recall** | **Accuracy [%]** | **F1-score** | **Precision** | **Recall** | **Accuracy [%]** | **F1-score** |
| *Alternaria* *alternata* | 0.71 | 0.42 | 41.6 | 0.53 | 0.74 | 0.61 | 61.1 | 0.67 |
| *Alternaria arborescens* | 0.78 | 0.69 | 68.8 | 0.73 | 0.76 | 0.75 | 75.4 | 0.76 |
| *Alternaria botrytis* | 0.43 | 0.46 | 46.3 | 0.45 | 0.43 | 0.48 | 48.3 | 0.46 |
| *Alternaria chartarum* | 0.42 | 0.54 | 53.7 | 0.47 | 0.40 | 0.54 | 54.2 | 0.46 |
| *Alternaria terricola* | 0.66 | 0.52 | 52.0 | 0.58 | 0.62 | 0.46 | 45.5 | 0.52 |
| *Botrytis cinerea* | 0.69 | 0.83 | 82.9 | 0.75 | 0.86 | 0.92 | 91.5 | 0.89 |
| *Chaetomium globosum* | 0.61 | 0.82 | 82.2 | 0.70 | 0.68 | 0.85 | 84.9 | 0.75 |
| *Cladosporium cladosporioides* | 0.37 | 0.62 | 62.2 | 0.46 | 0.74 | 0.81 | 80.9 | 0.78 |
| *Cladosporium herbarum* | 0.28 | 0.21 | 20.7 | 0.24 | 0.62 | 0.60 | 60.3 | 0.61 |
| *Cladosporium sphaerospermum* | 0.29 | 0.22 | 22.2 | 0.25 | 0.60 | 0.77 | 77.1 | 0.68 |
| *Curvularia caricae-papayae* | 0.92 | 0.95 | 94.8 | 0.93 | 0.89 | 0.95 | 95.2 | 0.92 |
| *Epicoccum nigrum* | 0.76 | 0.88 | 87.7 | 0.82 | 0.83 | 0.84 | 84.1 | 0.84 |
| *Exserohilum rostratum* | 0.87 | 0.85 | 84.7 | 0.86 | 0.90 | 0.87 | 86.7 | 0.88 |
| *Fusarium culmorum* | 0.77 | 0.86 | 86.4 | 0.81 | 0.91 | 0.95 | 94.5 | 0.93 |
| *Fusarium pseudocircinatum* | 0.27 | 0.23 | 22.5 | 0.25 | 0.64 | 0.65 | 64.7 | 0.64 |
| *Pithomyces* *chartarum* | 0.74 | 0.76 | 76.1 | 0.75 | 0.80 | 0.80 | 80.1 | 0.80 |
| *Stemphylium vesicarium* | 0.52 | 0.54 | 54.0 | 0.53 | 0.67 | 0.56 | 55.5 | 0.61 |
| **Macro average** | **0.59** | **0.61** | **61.1** | **0.60** | **0.71** | **0.73** | **72.9** | **0.72** |
| **Genus (combined species)** | **Precision** | **Recall** | **Accuracy [%]** | **F1-score** | **Precision** | **Recall** | **Accuracy [%]** | **F1-score** |
| *Alternaria* | 0.93 | 0.85 | 85.2 | 0.89 | 0.94 | 0.92 | 92.1 | 0.93 |
| *Botrytis* | 0.69 | 0.83 | 82.9 | 0.75 | 0.86 | 0.92 | 91.5 | 0.89 |
| *Chaetomium* | 0.61 | 0.82 | 82.2 | 0.70 | 0.68 | 0.85 | 84.9 | 0.75 |
| *Cladosporium* | 0.71 | 0.82 | 81.8 | 0.76 | 0.79 | 0.87 | 87.1 | 0.83 |
| *Curvularia* | 0.92 | 0.95 | 94.8 | 0.93 | 0.89 | 0.95 | 95.2 | 0.92 |
| *Epicoccum* | 0.76 | 0.88 | 87.7 | 0.82 | 0.83 | 0.84 | 84.1 | 0.84 |
| *Exserohilum* | 0.87 | 0.85 | 84.7 | 0.86 | 0.90 | 0.87 | 86.7 | 0.88 |
| *Fusarium* | 0.73 | 0.77 | 77.0 | 0.75 | 0.88 | 0.91 | 91.1 | 0.90 |
| *Pithomyces* | 0.74 | 0.76 | 76.1 | 0.75 | 0.80 | 0.80 | 80.1 | 0.80 |
| *Stemphylium* | 0.52 | 0.54 | 54.0 | 0.53 | 0.67 | 0.56 | 55.5 | 0.61 |
| **Macro average** | **0.75** | **0.81** | **80.6** | **0.77** | **0.82** | **0.85** | **84.8** | **0.83** |

**Supplementary Table 3**

Precision, recall, accuracy and F1-score values from classification with the Plair Rapid-E+ system. *Alternaria* spp. include the mixed species *A. alternata*, *A. arborescens*, *A. botrytis*, *A. chartarum* and *A. terricola*. *Cladosporium* spp. include the mixed species *Cladosporium cladosporioides*, *Cladosporium herbarum* and *Cladosporium sphaerospermum*.

| **Species** | **Precision** | **Recall** | **Accuracy [%]** | **F1-score** |
| --- | --- | --- | --- | --- |
| *Alternaria* spp. | 0.72 | 0.54 | 83.4 | 0.62 |
| *Botrytis cinerea* | 0.64 | 0.71 | 89.9 | 0.67 |
| *Cladosporium* spp. | 0.90 | 0.75 | 92.7 | 0.82 |
| *Curvularia caricae-papayae* | 0.56 | 0.87 | 95.1 | 0.68 |
| *Epicoccum nigrum* | 0.43 | 0.64 | 88.9 | 0.51 |
| *Exserohilum rostratum* | 0.49 | 0.43 | 87.7 | 0.46 |
| *Pithomyces* *chartarum* | 0.46 | 0.54 | 87.5 | 0.49 |
| **Macro average** | **0.60** | **0.64** | **89.3** | **0.61** |

**Supplementary Figure 1**

Macromorphology of fungi grown in Petri dishes with Potato Dextrose Agar medium (for five *Alternaria* species) or diluted Sabouraud medium (for the 12 other species), for about two weeks at 25°C in the dark.

**
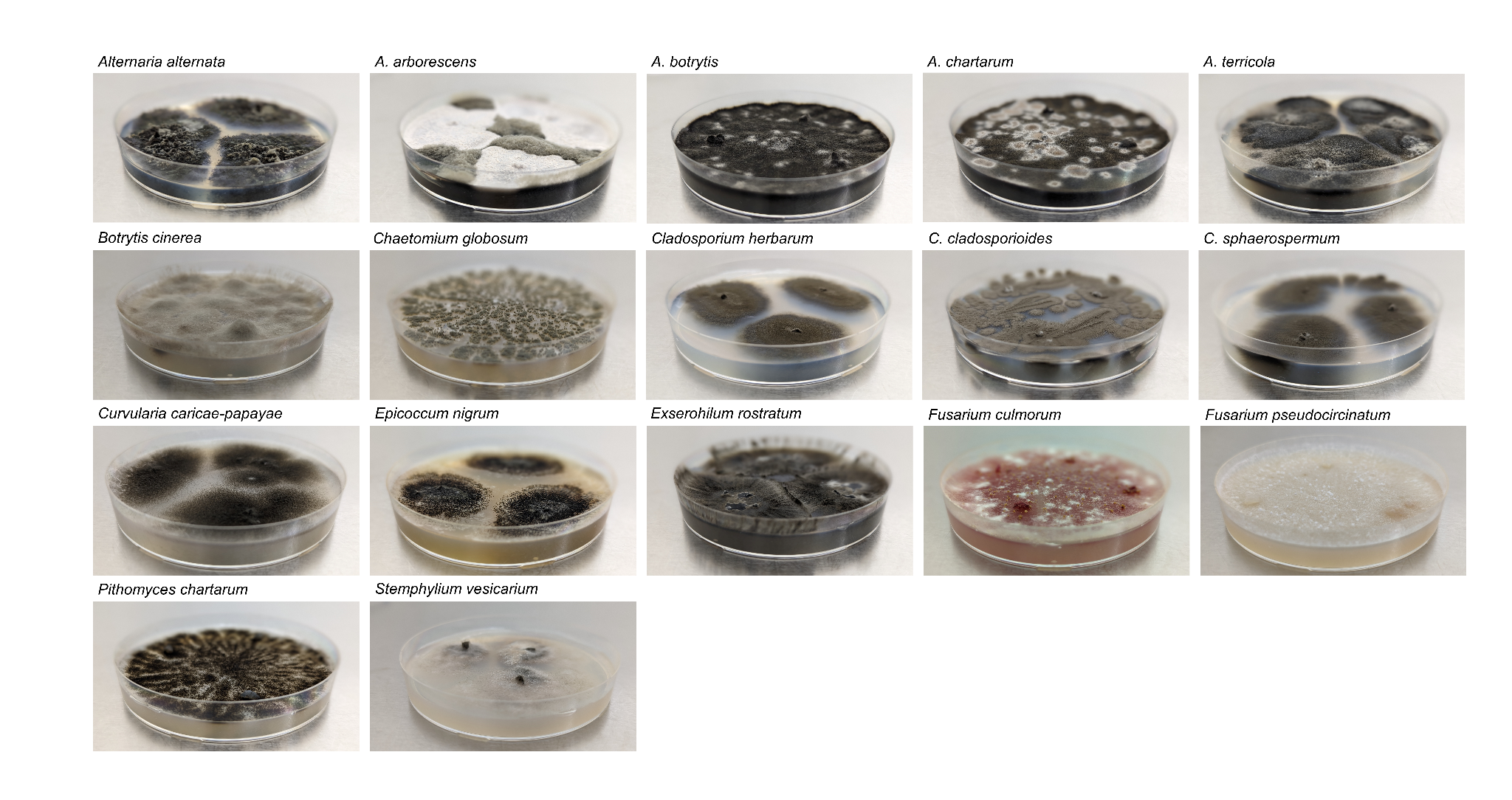
**

**Supplementary Figure 2**

Colony morphology as observed under stereomicroscope of fungi grown in Petri dishes with Potato Dextrose Agar medium (for five *Alternaria* species) or diluted Sabouraud medium (for the 12 other species), for about two weeks at 25°C in the dark.


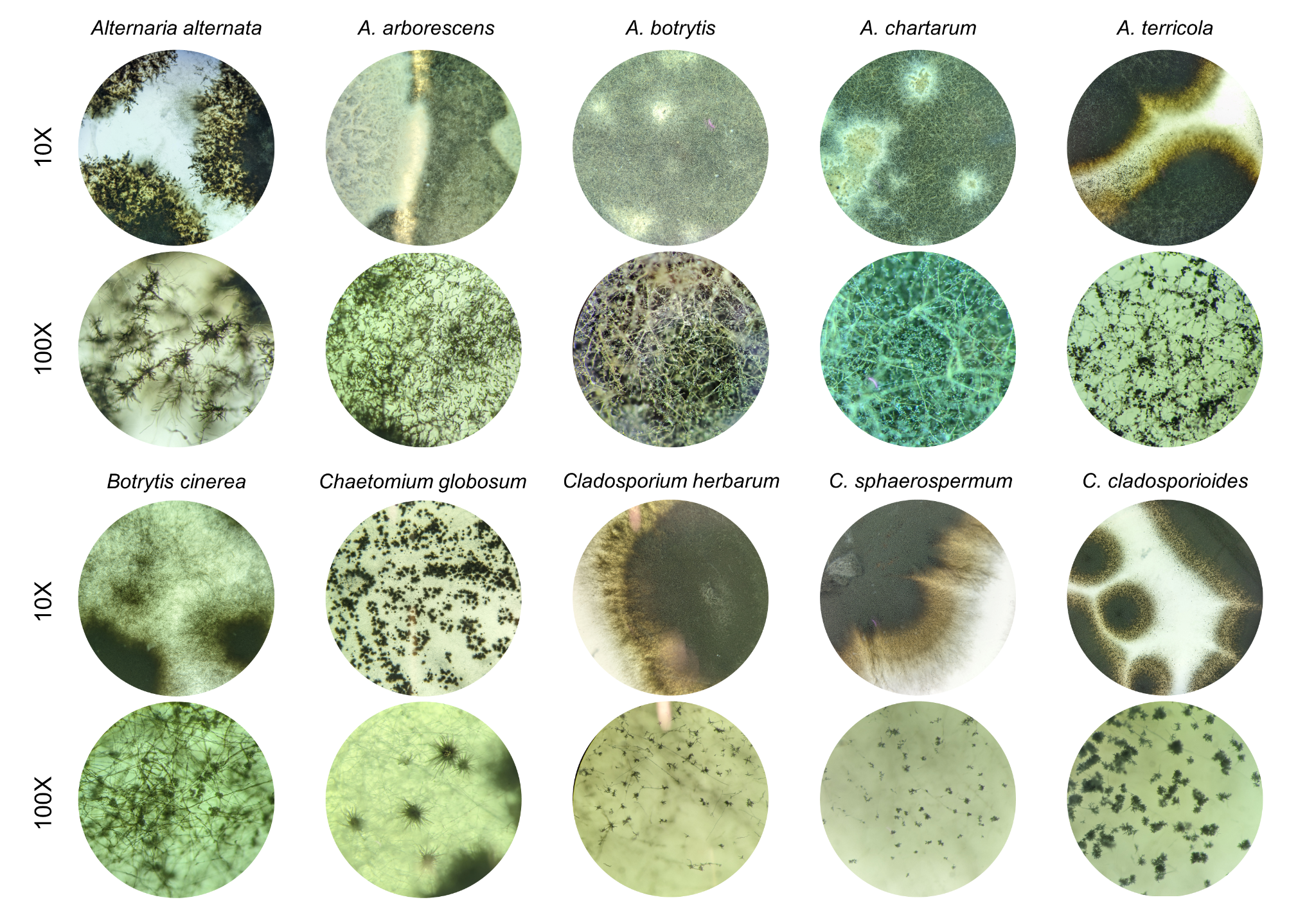


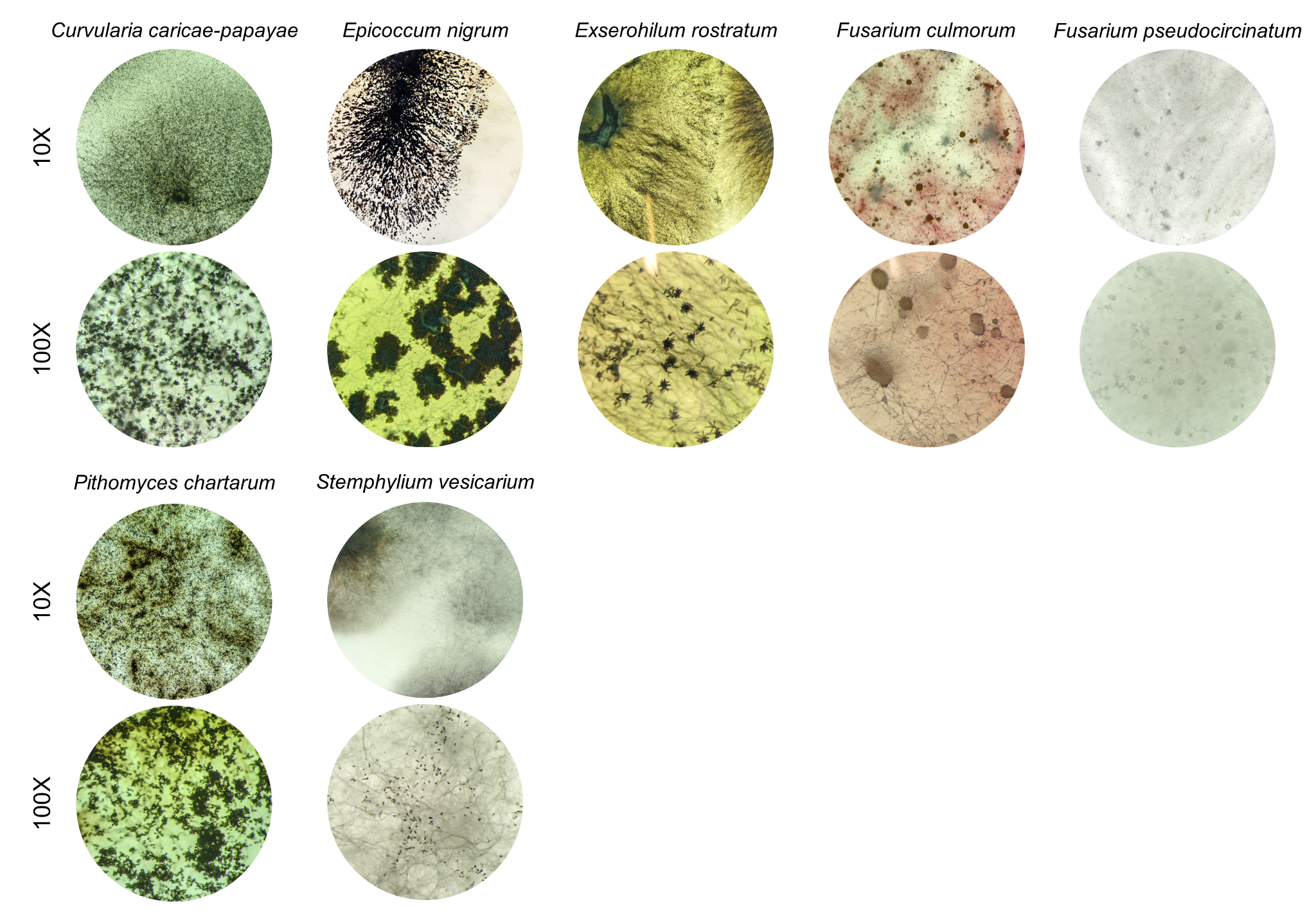


**Supplementary Figure 3**

Micromorphology and purity of spores harvested from the selected 17 fungal species cultures, as observed under light microscope. Dry samples were diluted in lactophenol cotton blue.


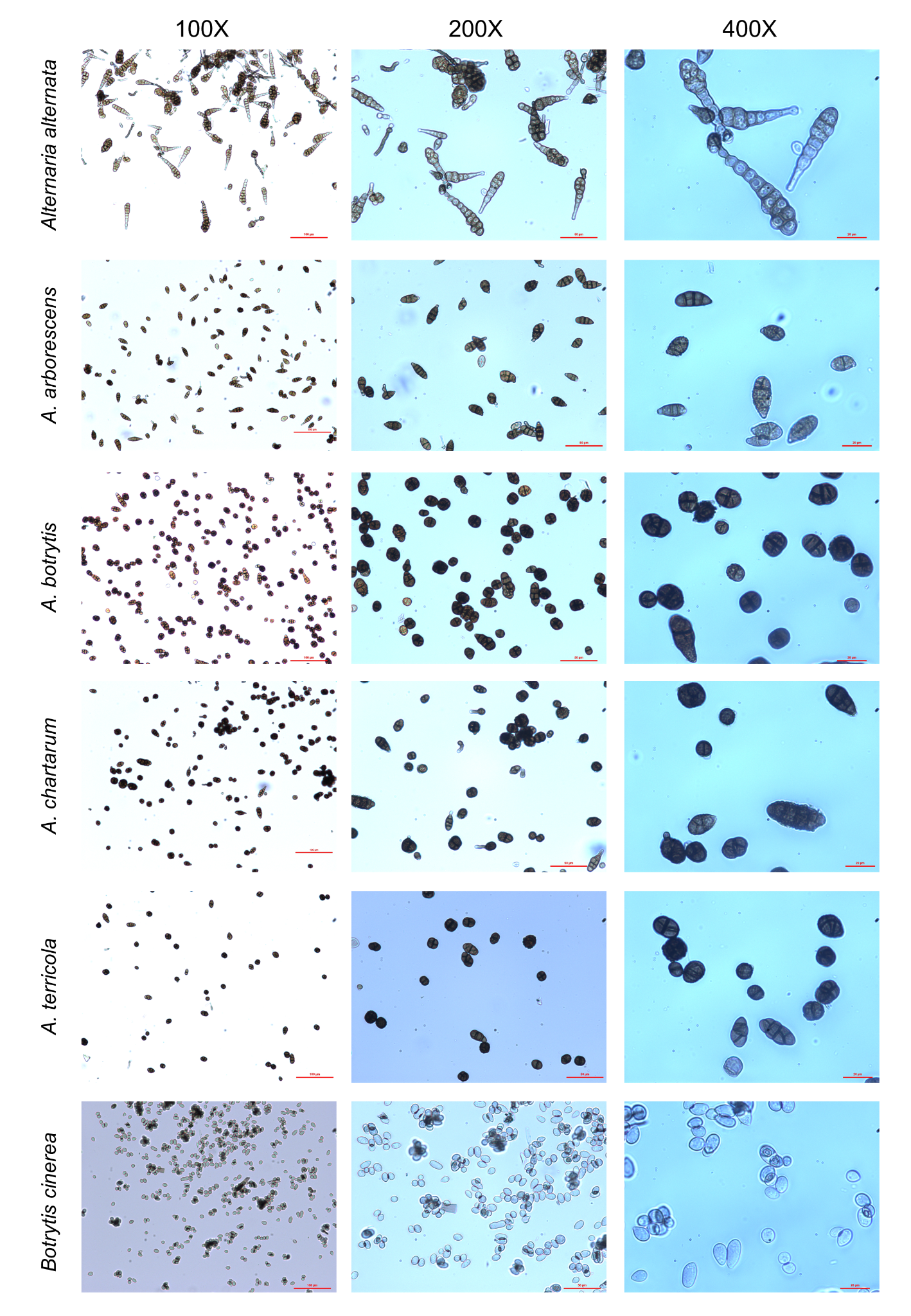


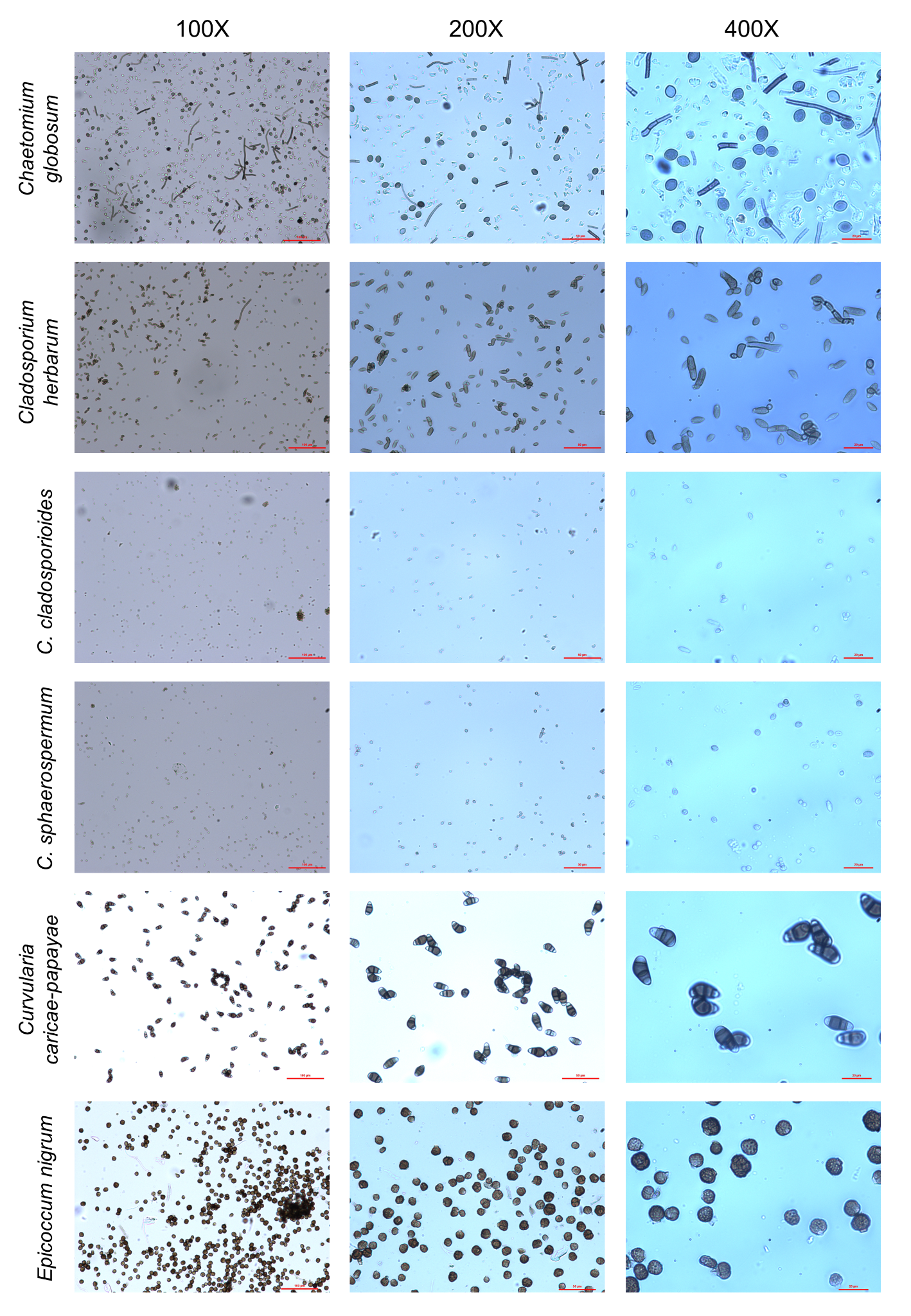


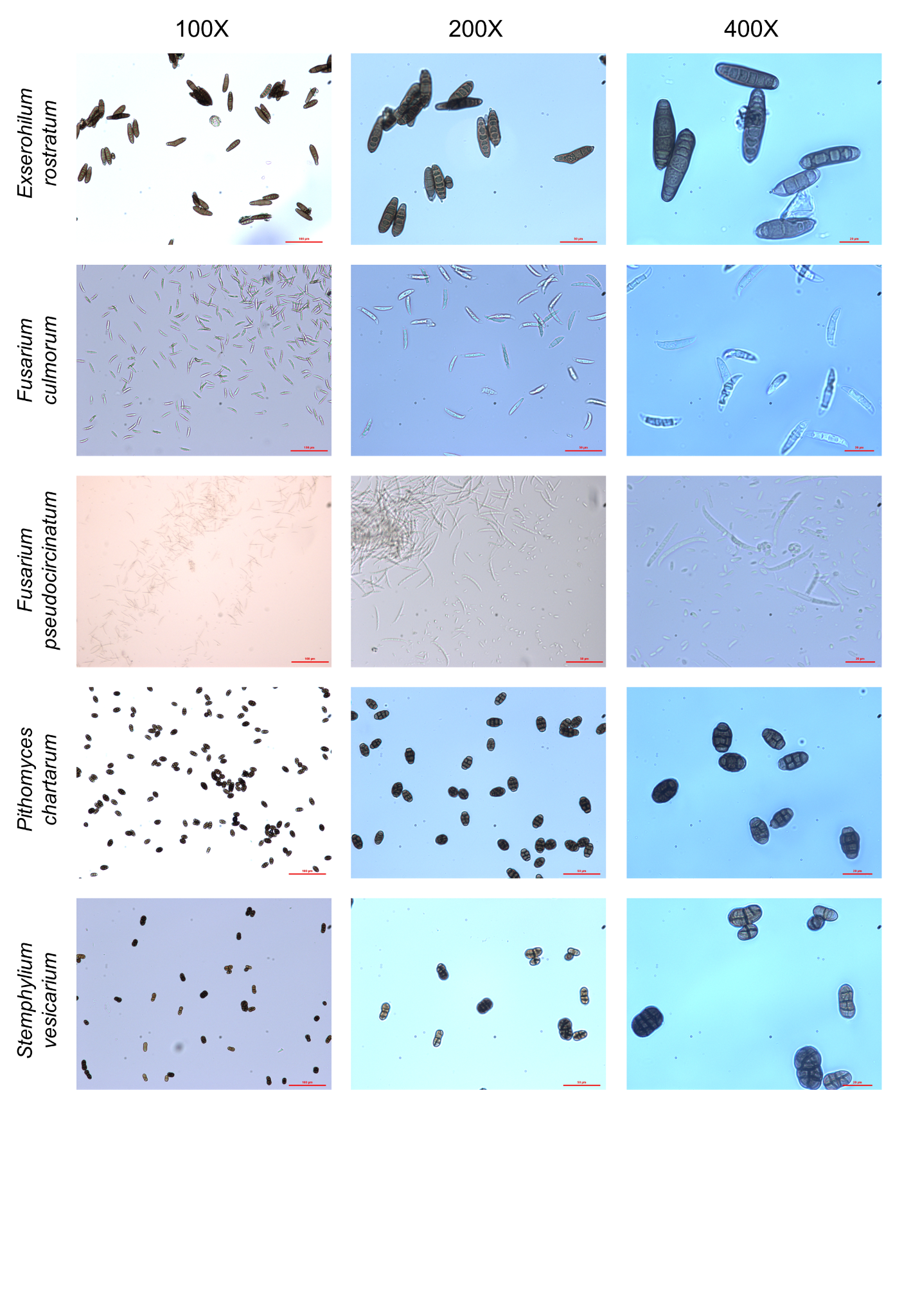


**Supplementary Figure 4**

Median relative fluorescence recorded by the SwisensPoleno Jupiter with standard deviation indicated by whiskers, for 17 selected fungal species.

**
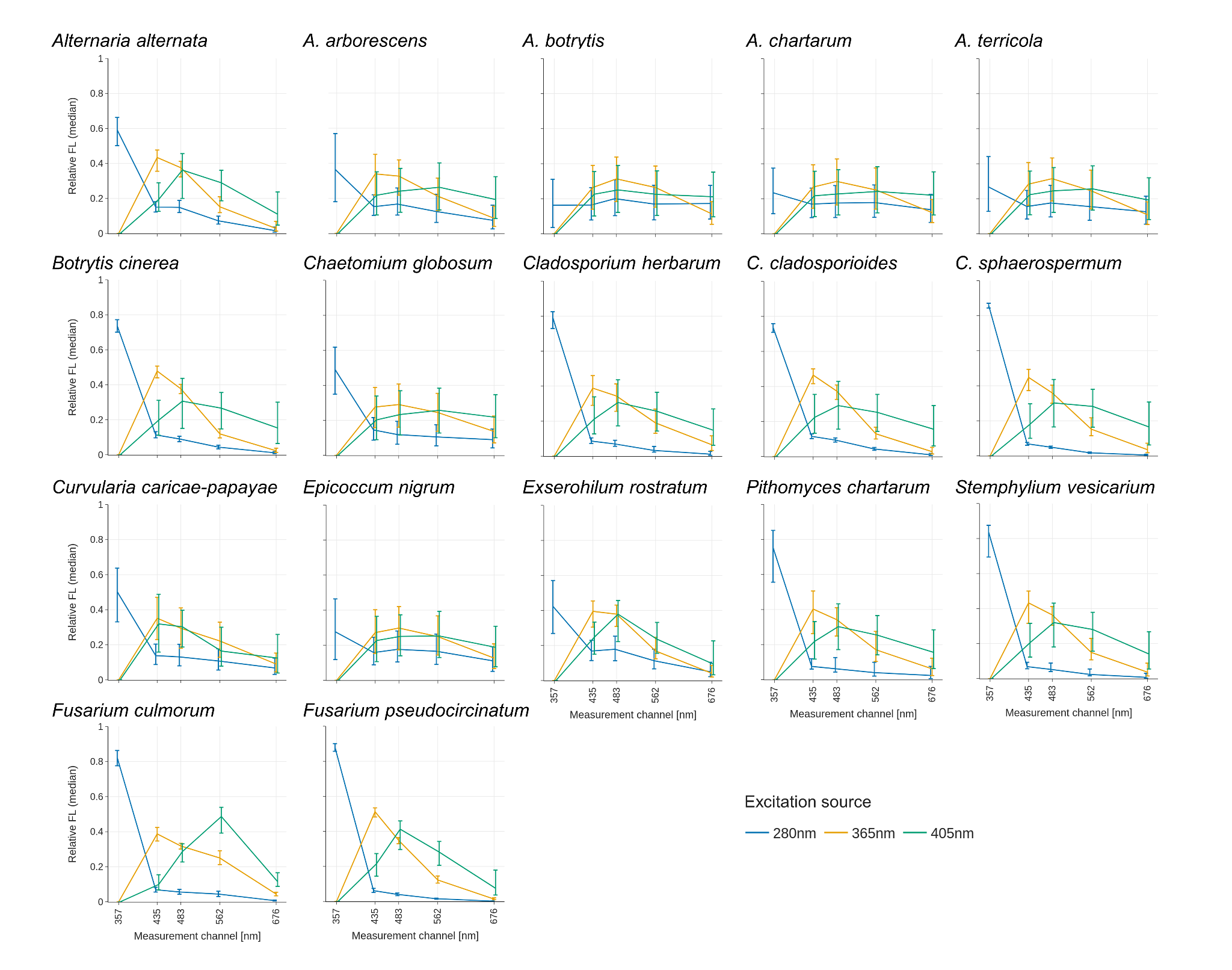
**

**Supplementary Video 1**

Dry spore harvesting of *Pithomyces chartarum* (strain IHEM 15222), grown on a Petri dish with diluted Sabouraud medium for 10 days at 25°C in the dark, using a cyclone collector.


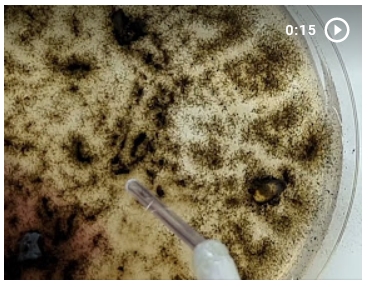


File uploaded under embargo on Zenodo:

Bruffaerts, N., Graf, E., Matavulj, P., Tiwari, A., Pyrri, I., Zeder, Y., Erb, S., Plaza, M., Dietler, S., Bendinelli, T., D'hooge, E., & Sikoparija, B. (2024, April 19). Supplementary Video 1. Zenodo. <https://doi.org/10.5281/zenodo.10998151>
